# Efficacy and Safety of Mifepristone in the Treatment of Male US Veterans With Posttraumatic Stress Disorder: A Phase 2a Randomized Clinical Trial

**DOI:** 10.1001/jamanetworkopen.2023.10223

**Published:** 2023-05-09

**Authors:** Julia A. Golier, Xue Li, Marcel Bizien, Robin A. Hurley, Brendan W. Bechard, Timothy Kimbrell, Janine D. Flory, Dewleen G. Baker, Rachel Yehuda, Domenic J. Reda

**Affiliations:** 1 Veterans Affairs (VA) Medical Center, Bronx, New York; 2 Department of Psychiatry, Mount Sinai School of Medicine, New York, New York; 3 VA Cooperative Studies Program, Hines, Illinois; 4 VA Cooperative Studies Program Clinical Research Coordinating Center, Albuquerque, New Mexico; 5 VA Medical Center, Salisbury, North Carolina; 6 Department of Psychiatry and Radiology, Wake Forest School of Medicine, Winston-Salem, North Carolina; 7 Central Arkansas VA Healthcare, University of Arkansas, Fayetteville; 8 VA San Diego Healthcare System, University of California, San Diego, San Diego

## Abstract

**Importance:**

To date, no psychopharmacologic treatment has been found to be uniformly effective in veterans with posttraumatic stress disorder (PTSD); novel targets and approaches are needed to treat this disabling disorder.

**Objective:**

To examine whether treatment with the glucocorticoid receptor antagonist mifepristone yields a signal for clinical efficacy in male veterans with PTSD.

**Design, Setting, and Participants:**

This phase 2a, double-blind, parallel-group randomized clinical trial was conducted from November 19, 2012 (accrual started), through November 16, 2016 (final follow-up), within the US Department of Veterans Affairs. Participants were male veterans with chronic PTSD and a screening Clinician-Administered PTSD Scale score of 50 or higher. A total of 181 veterans consented to participation. Statistical analysis was conducted between August 2014 and May 2017.

**Interventions:**

Participants were randomized in a 1:1 ratio to mifepristone (600 mg) or matched placebo taken orally for 7 days.

**Main Outcomes and Measures:**

The clinical outcome was whether a veteran achieved a clinical response status (a reduction of ≥30% of total Clinician-Administered PTSD Scale score from baseline) at 4- and 12-week follow-up. On the basis of a binary statistical selection rule, a difference in the proportion of treatment vs control group responders of 15% would be a clinically relevant difference. Self-report measures of PTSD and associated symptoms were also obtained. Neuroendocrine outcomes and plasma levels of mifepristone were measured. Safety was assessed throughout the study. The primary analysis was based on a multiple imputation technique to address missing outcome data; thus, some participant numbers may not appear as whole numbers.

**Results:**

A total of 81 veterans were enrolled and randomized. Excluding 1 participant randomized in error, 80 were included in the modified intention-to-treat analysis (41 randomized to mifepristone and 39 to placebo). The mean (SD) age was 43.1 (13.7) years. A total of 15.6 (38.1%) in the mifepristone group and 12.1 (31.1%) in the placebo group were clinical responders at 4 weeks in the analysis using the multiple imputation technique. The group difference in the proportion of clinical responders (7.0%) was less than the predefined margin of 15% indicating signal for clinical efficacy. In an exploratory analysis, the difference in response to mifepristone vs placebo in the subgroup with no lifetime history of traumatic brain injury (TBI) (7.0 [50.0%] vs 3.0 [27.3%]; difference, 22.7%) exceeded the efficacy margin at 4 weeks and was sustained at 12 weeks. In contrast, in veterans with PTSD and lifetime TBI, the response rate to mifepristone was lower than placebo at 12 weeks (7.4 [27.4%] vs 13.5 [48.3%]; difference, −20.9%).

**Conclusions and Relevance:**

This study did not detect a signal for efficacy for mifepristone at 600 mg/d for 1 week in male veterans with chronic PTSD. Thus, this study does not support a phase 3 trial in this population. Future studies of mifepristone for the treatment of PTSD may be of interest in those without a history of TBI or in samples with a low base rate of lifetime head trauma.

**Trial Registration:**

ClinicalTrials.gov Identifier: NCT01946685

## Key Points

**Question:**

Is there evidence of a signal for clinical efficacy for the glucocorticoid receptor antagonist mifepristone in the treatment of male veterans with posttraumatic stress disorder?

**Findings:**

In this randomized clinical trial of short-term mifepristone treatment (600 mg per day for 7 days) in 80 male veterans with posttraumatic stress disorder (41 receiving mifepristone and 39 receiving placebo), the difference in the clinical response rate to mifepristone vs placebo at 4 and 12 weeks was less than the predefined clinical margin used to define efficacy.

**Meaning:**

These results do not support an indication for a phase 3 trial of this regimen in this heterogeneous population.

## Teaser

This phase 2a randomized clinical trial compares the clinical response to mifepristone and placebo in male veterans with posttraumatic stress disorder to determine whether there is a signal for clinical efficacy.

## Introduction

Posttraumatic stress disorder (PTSD) is a common and frequently disabling psychiatric disorder often treated with psychopharmacologic agents. Most veterans with PTSD in the Veterans Administration (VA) are treated with psychotropics, most commonly selective serotonin reuptake inhibitors or serotonin-norepinephrine reuptake inhibitors, trazodone, prazosin, benzodiazepines, and antipsychotics.^1^ Clinical trials have not found selective serotonin reuptake inhibitors or other agents to be effective in veterans with PTSD,^2,3,4^ suggesting a need for a new approach.

Dysregulation of the hypothalamic-pituitary-adrenal (HPA) axis is associated with stress-related psychiatric disorders^5,6,7^ and viewed as a potential treatment target.^6,8,9^ Mifepristone is a steroid with antiglucocorticoid and antiprogesterone properties approved by the US Food and Drug Administration for medical abortion and Cushing disease. Mifepristone treatment in patients with Cushing disease led to rapid and sustained improvement in depression and psychosis.^9^ Subsequent short-term (1-week) trials of mifepristone in primary affective and psychotic disorders demonstrated clinical improvement with sustained efficacy.^9,10,11,12^ The outcomes of controlled trials have been mixed; successful outcomes in psychotic depression are associated with mifepristone levels greater than or equal to 1637 ng/mL.^13^

The proposed mechanism of action has been hypothesized to reflect a resetting of the HPA axis through the restoration of the balance of the corticosteroid receptors^6,8^ (glucocorticoid receptors [GRs] and mineralocorticoid receptors [MRs]), which mediate the initiation and containment of the stress response. Mifepristone blocks GRs but not MRs, leading to short-term increases in cortisol and adrenocorticotropic hormone (ACTH) levels due to the blockade of negative feedback inhibition. The occupancy of GRs and increased exposure of MRs to cortisol centrally are thought to downregulate MRs and reset the HPA axis.^6,8,10^

Owing to HPA axis dysregulation and enhanced GR sensitivity in PTSD,^14,15,16^ a similar 1-week regimen was tested in a small number of veterans with PTSD who showed substantial improvement at 1 month.^17^ In Gulf War illness, a condition with a similar profile of neuroendocrine dysfunction,^18^ mifepristone improved neuropsychological performance.^19^ Because this is the first significant trial in PTSD, an exploratory phase 2a trial for efficacy and safety was conducted.

The primary objective of this study was to compare the clinical response to mifepristone (600 mg/d for 1 week) and placebo in male veterans with PTSD to see if there is a signal (detected by ranking and selection statistical theory); a positive signal would justify hypothesis testing in a subsequent phase 3 trial. The secondary objectives were to characterize the trajectories of PTSD symptoms during the study and evaluate safety and tolerability. For descriptive purposes, the effects on associated clinical (depression, sleep quality, and anger) and laboratory (cortisol, ACTH, and mifepristone levels) parameters were characterized.

## Methods

### Study Design

This study was a 12-week, double-blind, placebo-controlled, parallel-group randomized clinical trial of a fixed dose of mifepristone in 5 VA outpatient clinics, conducted from November 19, 2012 (accrual started), through November 16, 2016 (final follow-up). The study was approved by each site’s institutional review board, conducted per good clinical practice guidelines, and monitored by an independent data monitoring committee. Written informed consent was obtained from every participant before enrollment. A complete list of the work group members can be found in the eAppendix in Supplement 1. A total of 181 veterans consented. This study followed the Consolidated Standards of Reporting Trials (CONSORT) reporting guideline. The trial protocol is available in Supplement 2.

This study was initially a 3-arm study, but the 1200-mg dose arm was removed after an unanticipated event (lethargy and bradycardia). Following a pause and review by the data monitoring committee, institutional review boards (eTable 3 in Supplement 1), Office of Research Oversight, and US Food and Drug Administration, enrollment resumed without this arm and with the addition of a safety check 48 to 72 hours after treatment initiation.

### Participants and Eligibility

Male veterans with PTSD precipitated by military trauma were eligible if their screening Clinician-Administered PTSD Scale (CAPS) total score was 50 or higher. Data on self-reported race and ethnicity were included for descriptive purposes and to assess for diversity. Exclusions included active suicidal ideation, current substance abuse, significant medical conditions, schizophrenia or bipolar disorder, and use of potent CYP3A4 inhibitors or oral corticosteroids. Initially, psychotropic medications were exclusionary, which proved to be a significant barrier to recruitment; veterans on stable doses were subsequently included. Assessments included CAPS,^20^ Structured Clinical Interview for *Diagnostic and Statistical Manual of Mental Disorders* (Fourth Edition), Ohio State University Traumatic Brain Injury Identification Method,^21^ Columbia Suicide Severity Rating Scale,^22^ the Miller Forensic Assessment of Symptoms Test,^23^ self-reports, and medical evaluations.

### Randomization and Blinding

Veterans were randomly assigned 1:1 to mifepristone or matched placebo (donated by Corcept Therapeutics) through a web-based randomization system stratified by site. Participants and all study personnel at the recruiting sites were blinded to treatment allocation.

### Intervention

Randomized veterans were assigned to take 600 mg of mifepristone or placebo for 7 days at bedtime and were prospectively followed up for up to 12 weeks (see the trial protocol in Supplement 2 for detailed timeline of assessments). Counts of returned medication were used to assess adherence.

### Study Outcomes

The primary outcome was clinical response status, defined as a 30% or greater reduction in total CAPS (past week) score from baseline to 4 weeks. On the basis of a binary statistical selection rule, an absolute difference in the proportion of treatment vs control group responders of 15% would be considered a clinically relevant difference.

The secondary outcome measures included the following: (1) proportion of clinical responders at 12 weeks, (2) trajectories of continuous CAPS scores, and (3) percentage of study drug–related adverse events. The descriptive outcomes included changes in (1) PTSD symptom subscale scores, (2) depressive symptoms (Beck Depression Inventory total score),^24^ (3) PTSD symptoms (PTSD Checklist total score),^25^ (4) sleep quality (Pittsburgh Sleep Quality Inventory total score),^26^ (5) anger (State-Trait Anger Expression Inventory total score),^27^ (6) functional impairment, and (7) plasma cortisol and ACTH levels and levels of mifepristone and its metabolites. No changes were made to the trial’s outcomes.

### Sample Size

Statistical selection theory was used. This method requires a much smaller sample size than traditional hypothesis testing when multiple doses are being evaluated, resulting in a reduction of both clinical costs and adverse events, and is used for phase 2 drug development to determine whether to pursue further clinical testing of a specific compound.^28,29,30^ The sample size was chosen to provide at least a 90% probability that the active treatment would be identified as superior if the absolute difference in the response rate between the active treatment and the placebo was at least 15%. This percentage was based on responder rate differences observed in previous positive trials in PTSD.^31,32,33^ Accounting for a 40% placebo response rate, 72 completers at 4 weeks were required; the sample was inflated to 80 to account for 10% attrition. There were no interim analyses or statistical stopping guidelines. This approach makes no attempt to control for type I error; the results generated from this study are intended to provide the basis for a larger, more definitive, phase 3 trial in which type I error and power are well controlled.

### Statistical Analysis

Baseline comparisons (Table 1) were tested using the Wilcoxon rank-sum test for age, the 2-tailed *t* test for age at focal trauma and combat experience scale, and Fisher exact test for all categorical variables. The primary analysis of the clinical responses was performed on a modified intent-to-treat (mITT) principle that included randomized participants who took study medication. Responses were estimated using multiple imputation techniques for missing CAPS scores at end points. The imputation was performed within each treatment group and used baseline variables, medication adherence, and available CAPS scores at other time points.^34^ Clinical responder rates were then combined from multiple imputed data sets. Because of this multiple imputation technique, some participant numbers may not appear as whole numbers.

**Table 1.** Baseline Participant Characteristics (Modified Intention-to-Treat Population)^a^

Characteristic	Mifepristone (n = 41)	Placebo (n = 39)
Demographic characteristics		
Age, mean (SD), y	43.7 (13.7)	42.4 (13.9)
Race		
African American or Black	14 (34.1)	11 (28.2)
American Indian/Alaska Native	1 (2.4)	0
Asian	1 (2.4)	0
Native Hawaiian/Pacific Islander	0	1 (2.5
White	20 (48.8)	26 (66.7)
Other^b^	5 (12.2)	1 (2.6)
Ethnicity		
Not Spanish, Hispanic, or Latino	29 (70.7)	25 (64.1)
Spanish, Hispanic, or Latino	12 (29.3)	14 (35.9)
Educational level		
High school or less	11 (26.8)	4 (10.3)
Some college or associate degree	22 (53.7)	27 (69.2)
College graduate or higher	8 (19.5)	8 (20.5)
Marital status		
Married or common law marriage	21 (51.2)	19 (48.7)
Separated, divorced, or widowed	15 (36.6)	10 (25.6)
Civil commitment or single	5 (12.2)	9 (23.1)
Missing	0	1 (2.6)
Employment status		
Currently working	18 (43.9)	10 (25.6)
Student	7 (17.1)	3 (7.7)
Unemployed or disabled	12 (29.3)	21 (53.8)
Retired	4 (9.8)	5 (12.8)
Household income >$50 000	10 (24.4)	12 (30.8)
Branch of military		
Army	23 (56.1)	19 (48.7)
Marine Corps	9 (22.0)	9 (23.1)
Navy	7 (17.1)	2 (5.1)
Air Force	1 (2.4)	2 (5.1)
National Guard	1 (2.4)	2 (5.1)
Multiple branches	0	5 (12.8)
Service period		
Vietnam Conflict (August 1964-April 1975)	5 (12.2)	5 (12.8)
May 1975-July 1990	5 (12.2)	2 (5.1)
Persian Gulf War (August 1990-February 1991)	4 (9.8)	5 (12.8)
March 1991-September 2001	2 (4.9)	0
Afghanistan/Iraq Conflict (Oct 2001-present)	22 (53.7)	23 (59.0)
Multiple services	3 (7.3)	4 (10.3)
Focal trauma assessment		
Age at focal trauma, mean (SD), y	24.7 (6.7)	24.7 (9.5)
Combat experience scale score, mean (SD)	53.9 (14.4)	58.0 (13.5)
Focal trauma related to PTSD from life events checklist		
Combat or war zone exposure	32 (78.0)	31 (79.5)
Other	9 (22.0)	8 (20.5)
TBI assessment		
Type of head injury		
No injury	11 (26.8)	8 (20.5)
Improbable TBI	3 (7.3)	3 (7.7)
Possible TBI	4 (9.8)	4 (10.3)
Mild TBI	19 (46.3)	19 (48.7)
Moderate TBI	4 (9.8)	4 (10.3)
Severe TBI	0	1 (2.6)
TBI^c^	27 (65.9)	28 (71.8)
Currently experiencing symptoms from past TBI	6 (14.6)	7 (17.9)
Lifetime comorbid psychiatric conditions measured by Structured Clinical Interview for DSM-IV Disorder		
Mood disorder	29 (70.7)	26 (66.7)
Bipolar II disorder	1 (2.4)	2 (5.1)
Depressive disorder	26 (63.4)	24 (61.5)
Dysthymia disorder	3 (7.3)	1 (2.6)
Substance abuse disorder	27 (65.9)	23 (59.0)
Alcohol abuse or dependence	25 (61.0)	23 (59.0)
Drug abuse or dependence	6 (14.6)	10 (25.6)
Anxiety disorder	16 (39.0)	9 (23.1)
One or more comorbid mental disorder	37 (90.2)	33 (84.6)

Abbreviations: DSM-IV, *Diagnostic and Statistical Manual*, 4th edition; TBI, traumatic brain injury.

^a^ Data are presented as number (percentage) of study participants unless otherwise indicated.

^b^ Other includes individuals of multiple races.

^c^ Participants were considered as having no TBI if they reported no head injury or improbable TBI per the Ohio State University TBI assessment form. Participants were classified as having TBI if they reported any degree of TBI following head trauma, ranging from possible to severe.

As secondary analyses, 90% CIs for the difference in response rates were provided, and logistic regression models adjusting for baseline characteristics were developed. Sensitivity analyses for handling missing CAPS scores included complete case analysis, worst case scenario, and last available case carried forward.

Additional analyses of the CAPS score trajectory over time using repeated-measures covariance-pattern models and allowing arbitrary patterns in the mean response and covariance structure were conducted.^35^ Covariance-pattern models were also developed for repeatedly measured descriptive clinical outcomes assuming normally distributed data with the baseline value as one of the repeated measures, main effects for group and time, a group × time interaction term, and unstructured covariance matrix to account for correlated responses.

For all descriptive clinical outcomes, degrees of freedom were based on the Kenward-Roger method. Reported change values and 95% CIs are the least-squares estimates using the restricted maximum likelihood method for estimation. Functional impairment was analyzed using a mixed-effects proportional odds model with a random intercept. The primary outcome was also explored for subgroups defined by classification before randomization for PTSD severity, depression, lifetime traumatic brain injury (TBI) status, Miller Forensic Assessment of Symptoms Test scores, and psychotropic medication use.

For cortisol and ACTH levels, medians and IQRs were reported for the baseline values and the change values (within group) from baseline. Hodges-Lehmann estimates of median difference and 95% CIs were reported for the change values (between-group comparisons). *P* values are from nonparametric Wilcoxon rank-sum tests for between-group comparisons. The numbers of participants analyzed in the mifepristone group were 41 at 1 week and 38 at 4 weeks. The numbers of participants analyzed in the placebo group were 38 at 1 week and 35 at 4 weeks.

Treatment-emergent adverse events (TEAEs) reported by at least 4% of participants from either intervention group were compared. The numerator is the number of participants with the specified adverse event in each treatment arm. The denominator is the total number of participants within each arm in the safety analysis population. The Fisher exact test was used, except for the total number of TEAEs and number of TEAEs in the first week, for which the Pearson χ^2^ test was used.

Analyses were conducted between August 2014 and May 2017. SAS/STAT, version 9.4 (SAS Institute Inc) was used for statistical analysis. Hypothesis tests were 2-sided with an α = .05.

## Results

### Recruitment and Baseline Characteristics

**Figure 1.**
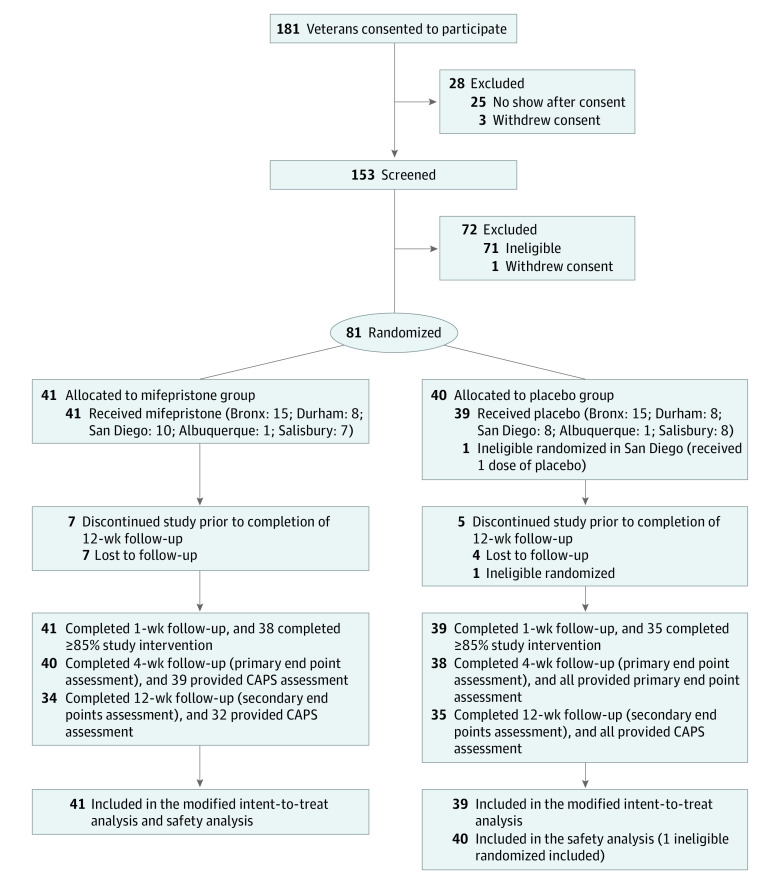
Flow Diagram of Participant Enrollment CAPS indicates Clinician-Administered PTSD Scale.

As shown in the flow diagram (Figure 1), of 181 veterans who consented, 81 were randomized (41 to mifepristone and 40 to placebo). The most common reasons for screen failures were CAPS score less than 50 (45 [25.7%]), not meeting PTSD criteria (22 [12.6%]), psychiatric medication exclusion (8 [10.3%]), diabetes or other significant medical conditions (9 [5.1%]), and current substance misuse (8 [4.6%]). Because 1 participant was randomized in error (had an exclusionary laboratory value), 80 were included in the mITT analysis; all were included in safety analyses.

The baseline participant demographic and clinical characteristics did not differ by group (Table 1). The mean (SD) age was 43.1 (13.7) years. Twenty-five participants (32.3%) were African American or Black, 46 (57.5%) were White, and 9 (11.3%) were of other race or ethnicity; 54 (67.5%) were non-Spanish, Hispanic, or Latino; and 26 (32.5%) were Spanish, Hispanic, or Latino. A majority of participants served during the Iraq and Afghanistan conflicts, had a history of TBI, had another psychiatric disorder, and had combat or war zone exposure as the precipitant to their PTSD.

### Treatment Adherence

Seventy participants completed the entire regimen, and 11 were lost to follow-up (7 in the mifepristone group and 4 in the placebo group). The numbers of participants with at least 85% treatment adherence were similar among the 2 groups (Figure 1). Those who took fewer than 12 tablets cited forgetfulness or adverse events (2 in each arm). No participant took fewer than 2 tablets.

### Clinical Outcomes

**Figure 2.**
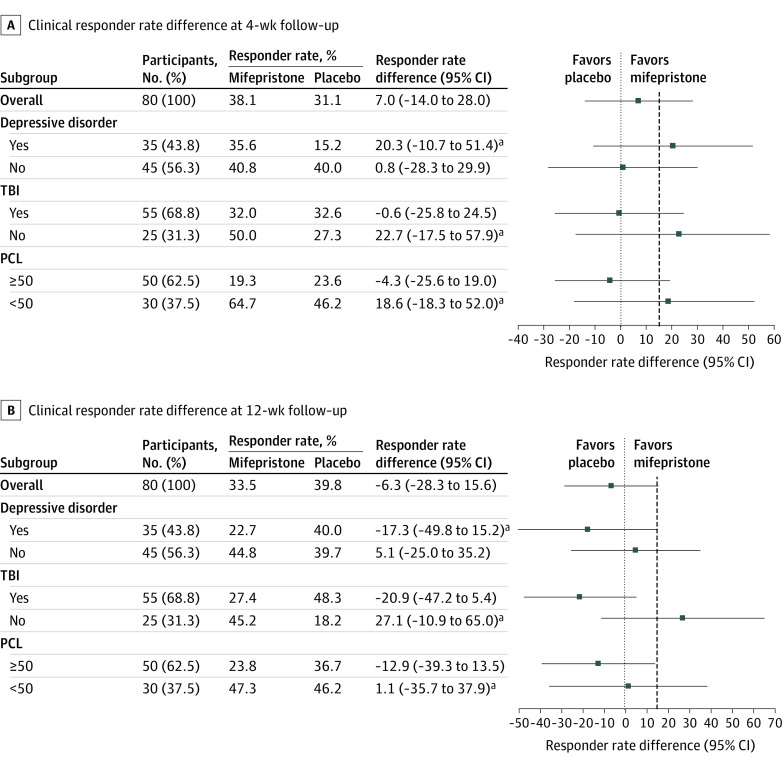
Clinical Responder Rate Differences Between Mifepristone and Placebo at 4 Weeks (Primary End Point) and 12 Weeks (Secondary End Point) Imputation was performed within treatment groups and used baseline variables, medication adherence rate, and available total Clinician-Administered PTSD Scale (CAPS) scores at other time points. The *P* value for the interaction of TBI by treatment is .25 at 12 weeks. The *P* values for the PTSD Checklist (PCL; ≥50 vs <50) by treatment interaction and depressive disorder by treatment interactions are, respectively, .33 and .32 at 4 weeks and 0.31 and 0.51 at 12 weeks. The vertical dashed line represents the predefined margin of 15% indicating efficacy. TBI indicates traumatic brain injury. ^a^Exact unconditional 95% CI for the difference of proportions.

At 4 weeks, more participants in the mifepristone (15.6 [38.1%]) than the placebo (12.1 [31.1%]) group were responders (Figure 2A). At 12 weeks, a lower responder rate in the mifepristone group (15.5 [33.5%]) than the placebo group (13.7 [39.8%]) was observed (Figure 2B). The response rate differences, 7.0% at 4 weeks and −6.3% at 12 weeks, were less than the predefined clinical efficacy margin (15%), implying no efficacy signal for mifepristone overall. The 95% CIs for responder rate difference at 4 weeks (−14% to 28%) and 12 weeks (−28.3% to 15.6%) and results of other methods to treat missing data all include zeros (eTable 1 in Supplement 2).

A significant subgroup × treatment interaction was found for the TBI vs no TBI subgroup at 12 weeks in the Breslow-Day test for treatment × subgroup interaction analysis (Figure 2A and B). The subgroup with no lifetime history of TBI (n = 25) showed a greater response to mifepristone than placebo (7.0 [50.0%] vs 3.0 [27.3%]; difference, 22.7%) at 4 weeks that was sustained at 12 weeks (6.3 [45.2%] vs 2.0 [18.2%]; difference, 27.1%). Both differences exceeded the defined threshold for clinical relevance. Because of the small sample size in this subgroup, neither signal achieved statistical significance. The 95% CIs for the response difference were −17.5% to 57.9% at 4 weeks and −10.9% to 65.0% at 12 weeks.

In those with lifetime TBI (n = 55), the responses to mifepristone and placebo were similar at 4 weeks (8.6 [32.0%] vs 9.1 [32.6%]). At 12 weeks, the response to mifepristone (7.4 [27.4%]) was lower than the response to placebo (13.5 [48.3%]; difference, −20.9%) (Figure 2B). The 95% CI for this difference was −47.2% to 5.4%, and the group difference was not significant (*P* = .12).

Mean total CAPS scores decreased over time in a curvilinear manner, and the reductions within each group were all significant (Table 2; eFigure 1 in Supplement 2); however, no significant between-treatment differences were observed at 4 weeks (difference, 1.9; 95% CI, −6.2 to 9.9; *P* = .65) and 12 weeks (difference, 2.9; 95% CI, −7.2 to 13.0; *P* = .57). On the basis of repeated-measures analysis (Table 2), there was no significant treatment group difference or interaction between time and group.

**Table 2.** Secondary and Descriptive Outcome Measures (Modified Intention-to-Treat Population)^a^

Outcome	Baseline		Change from baseline to 4 wk^b^			Change from baseline to 12 wk^c^		
	No.	Mean (SD)	Within-treatment difference, mean (95% CI)	Between-treatment difference		Within-treatment difference, mean (95% CI)	Between-treatment difference	
				Mean (95% CI)	*P* value		Mean (95% CI)	*P* value
**Secondary outcome**								
CAPS total score								
Mifepristone	41	67.0 (17.5)	−13.96 (−19.61 to −8.31)	1.87 (−6.19 to 9.93)	.65	−15.15 (−22.34 to −7.96)	2.91 (−7.20 to 13.01)	.57
Placebo	39	73.3 (16.7)	−15.83 (−21.57 to −10.08)			−18.06 (−25.16 to −10.96)		
**Descriptive clinical outcomes**								
CAPS intrusive symptom subscale score								
Mifepristone	41	14.7 (8.7)	−4.27 (−6.89 to −1.64)	1.23 (−2.52 to 4.98)	.52	−4.77 (−7.91 to −1.63)	3.01 (−1.39 to 7.41)	.18
Placebo	39	18.3 (20.0)	−5.49 (−8.17 to −2.82)			−7.78 (−10.86 to −4.70)		
CAPS avoidance symptom subscale score								
Mifepristone	41	29.0 (8.0)	−6.24 (−9.24 to −3.24)	0.57 (−3.70 to 4.85)	.79	−6.30 (−9.99 to −2.60)	−0.08 (−5.27 to 5.11)	.98
Placebo	39	30.8 (8.2)	−6.81 (−9.86 to −3.76)			−6.22 (−9.87 to −2.57)		
CAPS hyperarousal symptom subscale score								
Mifepristone	41	23.4 (5.4)	−3.35 (−5.16 to −1.53)	0.14 (−2.45 to 2.73)	.92	−3.22 (−5.37 to −1.08)	0.93 (−2.07 to 3.92)	.54
Placebo	39	24.2 (6.5)	−3.49 (−5.33 to −1.64)			−4.15 (−6.24 to −2.05)		
BDI total score								
Mifepristone	41	21.6 (9.0)	−3.50 (−6.20 to −0.79)	1.02 (−2.85 to 4.89)	.60	−2.25 (−5.34 to 0.83)	2.82 (−1.56 to 7.20)	.20
Placebo	39	22.8 (10.5)	−4.52 (−7.29 to −1.75)			−5.07 (−8.19 to −1.96)		
PCL total								
Mifepristone	41	52.8 (12.3)	−6.84 (−9.90 to −3.78)	1.80 (−2.59 to 6.20)	.42	−6.84 (−10.54 to −3.14)	1.28 (−4.01 to 6.57)	.63
Placebo	39	57.0 (12.8)	−8.65 (−11.80 to −5.49)			−8.12 (−11.90 to −4.34)		
PSQI total score								
Mifepristone	41	14.2 (3.2)	−1.63 (−2.67 to −0.59)	−0.36 (−1.85 to 1.13)	.63	−0.86 (−2.10 to 0.40)	1.19 (−0.58 to 2.96)	.19
Placebo	39	14.5 (3.5)	−1.27 (−2.34 to −0.21)			−2.04 (−3.29 to −0.79)		
STAXI total score								
Mifepristone	41	22.1 (8.2)	−0.61 (−2.78 to 1.56)	1.22 (−1.89 to 4.32)	.44	−1.73 (−3.85 to 0.39)	1.47 (−1.53 to 4.47)	.33
Placebo	39	22.1 (7.6)	−1.83 (−4.05 to 0.40)			−3.20 (−5.32 to −1.08)		
**Descriptive neuroendocrine outcomes** ^b^ ^,^ ^c^								
Plasma cortisol, μg/dL								
Mifepristone	41	14.7 (9.6 to 17.1)	22.3 (3.3 to 33.2)	20.6 (14.5 to 26.6)	<.001	−0.6 (−7.0 to 3.1)	−1.1 (−4.6 to 1.8)	.45
Placebo	38	13.2 (9.5 to 17.1)	−0.9 (−3.4 to 3.6)			−0.4 (−2.4 to 3.4)		
ACTH, μg/dL								
Mifepristone	41	25.5 (14.5 to 36.2)	28.8 (7.9 to 63.7)	28.9 (16.7 to 45.3)	<.001	−0.8 (−7.3 to 2.6)	−2.5 (−6.5 to 1.2)	.15
Placebo	38	21.7 (17.1 to 33.2)	−0.5 (−5.0 to 5.4)			1.5 (−5.5 to 6.2)		

Abbreviations: CAPS, Clinician-Administered PTSD Scale; BDI, Beck Depression Inventory; PCL, PTSD Checklist; PSQI, Pittsburgh Sleep Quality Inventory; STAXI, State-Trait Anger Expression Inventory.

SI conversion factors: To convert cortisol to nanomoles per liter, multiply by 27.588; ACTH to picomoles per liter, multiply by 0.22.

^a^ The CAPS total score (past week symptoms) has a possible range of 0 to 136, with higher scores indicating worse PTSD symptoms overall. The CAPS intrusive symptom subscale scores range from 0 to 40, the CAPS avoidance symptom total scores range from 0 to 56, and the CAPS hyperarousal symptom total scores range from 0 to 40, with higher scores indicating worse PTSD symptoms in the corresponding functional domains. Total BDI scores range from 0 to 63, with higher scores indicating more severe depression symptoms. Totals from the PCL range from 17 to 85, with higher scores indicative of more severe PTSD. The PSQI total scores range from 0 to 21, with higher scores indicating worse sleep quality. Negative values for changes over time for the within-group and between-group comparisons indicate improvement in functioning or quality of life. The score ranges are not based on the collected sample; instead, they are based on the standard questionnaire in general.

^b^ For the descriptive neuroendocrine outcomes, data are for the change from baseline to 1 week.

^c^ For the descriptive neuroendocrine outcomes, data are for the change from baseline to 4 weeks.

### Neuroendocrine and Mifepristone Level Outcomes

Cortisol and ACTH levels increased with active treatment; the change in levels from baseline to 1 week differed significantly between the groups (Table 2). However, the levels at week 4 were similar to baseline levels in both groups, and there was no significant between-group difference in the change from baseline to week 4.

In the mifepristone group only, responders (n = 15) vs nonresponders (n = 23) did not differ significantly in posttreatment median cortisol (13.2 vs 13.3 μg/dL [to convert to nanomoles per liter, multiply by 27.588]) or ACTH (median, 29.7 vs 22.1 pg/nL [to convert to picomoles per liter, multiply by 0.22]) levels. Within the entire sample, respondents had higher, but not significantly different, posttreatment ACTH and cortisol levels than nonresponders. When extreme ACTH outliers (1 in each group) were removed (eFigure 2 in Supplement 2), the posttreatment ACTH levels at 1 week were significantly higher in responders than nonresponders (median difference, 12.3; 95% CI, 0.5-27.3; *P* = .04).

Compared with nonresponders, mifepristone responders showed lower median levels of mifepristone (1305 vs 1610 ng/mL, *P* = .11) and all 3 metabolites. The largest difference was for metabolite RU-42633 (1485 vs 2100 ng/mL, *P* = .04) (eTable 2 in Supplement 2).

### Safety

Fourteen participants (34.1%) in the mifepristone group and 13 (32.5%) in the placebo group reported at least 1 study drug–related adverse event or possibly TEAE (Table 3). There was no significant difference between the 2 groups. Overall, mifepristone was well tolerated.

**Table 3.** Study Drug–Related or Possibly Related Adverse Events (Safety Population)

Adverse event	Mifepristone (n = 41)	Placebo (n = 40)	Total (N = 81)
No. (%) of participants with TEAEs	14 (34.1)	13 (32.5)	27 (33.3)
Adverse event severity			
No. of participants (No. of mild [grade 1] TEAEs)	13 (20)	12 (21)	25 (41)
No. of participants (No. of moderate [grade 2] TEAEs)	2 (4)	1 (1)	3 (5)
No. of participants (No. of severe [grade 3] TEAEs)	0	0	0
Adverse events onset			
No. (%) of participants [No. of events] before week 1	11 (26.5) [15]	11 (27.5) [17]	22 (27.2) [32]
No. (%) of participants [No. of events] between weeks 1 and 4	5 (12.2) [7]	4 (10.0) [5]	9 (11.1) [12]
No. (%) of participants [No. of events] between weeks 4 and 12	2 (4.9) [2]	0	2 (2.5) [2]
No. (%) of participants by body system affected (≥4% for any group)			
General disorder: fatigue	3 (7.3)	2 (5.0)	5 (6.2)
Skin disorder: rash or rash pruritic	2 (4.9)	3 (7.5)	5 (6.2)
Skin disorder: pruritus	2 (4.9)	0	2 (2.5)
Nervous system disorder: headache	2 (4.9)	4 (10.0)^a^	6 (7.4)
Nervous system disorder: dizziness	2 (4.9)	0	2 (2.5)
Gastrointestinal disorder: dry month	2 (4.9)	0	2 (2.5)

Abbreviation: TEAE, treatment-emergent adverse event.

^a^ Includes 1 exertional headache.

## Discussion

Direct manipulation of the HPA axis with mifepristone led to the anticipated short-term changes in neuroendocrine activity in this sample of veterans with PTSD and was well tolerated. However, the patterns of clinical response did not demonstrate a signal for efficacy at 4 or 12 weeks for mifepristone. Additionally, there were no mifepristone-related improvements in PTSD or associated symptoms measured continuously or longitudinally. As such, this trial did not provide a basis for a phase 3 trial of this treatment regimen in a heterogeneous population of male veterans with PTSD.

Cortisol and ACTH level increased in the mifepristone group but did not differ between responders and nonresponders in the mifepristone group. However, posttreatment ACTH levels were higher in responders than nonresponders for the entire sample, including those taking placebo. These results point to a possible relationship between improvement and change in neuroendocrine activity but include changes that are not limited to direct drug effects.

Lifetime TBI status was assessed in recognition of this signature injury of the wars in Iraq and Afghanistan and a contributor to postdeployment health outcomes.^36,37^ Most participants reported a lifetime history of head injury with sequelae that indicated likely TBI. Almost all participants also had another psychiatric disorder other than PTSD; most met the criteria for a mood disorder and a lifetime history of substance use disorder. Given this population’s clinical complexity and heterogeneity, subgroup analyses were undertaken.

Only TBI emerged as a relevant subgroup. In this exploratory analysis, veterans with no TBI demonstrated an efficacy signal at 4 and 12 weeks. In contrast, in veterans with TBI, there was a signal that favored placebo at 12 weeks. Given the broad CIs and small samples in these exploratory analyses, the potential efficacy signal of the no TBI subgroup should not be overstated. However, it raises the possibility that further study of mifepristone may be warranted in this subgroup or a PTSD population with a low base rate of head trauma. Given the opposing effects of mifepristone based on this categorization, TBI clinical assessments should be included in future psychopharmacologic trials of veterans with PTSD because it may influence the outcome.

### Limitations

This study has some limitations. Notably, it applies only to men. Insofar as mifepristone is an abortifacient, the risk profile would be different in women owing to its antiprogesterone effects. A separate study in women with PTSD accounting for these risks may be warranted.

This study was restricted to a single dose (600 mg) owing to the termination of the 1200-mg dose arm following an unanticipated event. A higher dose or longer duration of treatment might have yielded more participants with a better clinical response. If future studies are conducted, a broader range of doses should be considered to allow for a wider range of mifepristone levels and determine whether there is a relationship between drug level and outcome in PTSD.

This sample may not be generalizable to the larger population of treatment-seeking veterans in the VA system. More than half were excluded because of stringent medical and concomitant medication exclusions. Additionally, there was a high prevalence of lifetime history of TBI. Given the poor response in veterans with TBI to mifepristone, this may have contributed to the lack of efficacy in the overall trial.

## Conclusions

The results of this phase 2a randomized clinical trial do not support the indication for a phase 3 trial of this regimen in male veterans with chronic PTSD. A potential signal for efficacy was detected in the group without TBI, which should be considered exploratory.

## Notes

Supplement 1. **eAppendix.** CCTA #0004 Group Information **eTable 1.** Sensitivity Analyses of Clinical Response Rate (30% Reduction in CAPS Total Score) at 4 Weeks (Primary Endpoint) and 12 Weeks (Secondary Endpoint) **eTable 2.** Mifepristone Plasma and Metabolites at the End of 1-Week Dosing Phase by Clinical Responder Status (Limited to Mifepristone Group Only) **eFigure 1.** Plots of Estimated Means of Total CAPS Score Over Time **eFigure 2.** Plots of Cortisol and ACTH over Time by 4-Week Clinical Responder Status **eTable 3. **Institutional Review Boards

Supplement 2.
Trial Protocol

Supplement 3.
Data Sharing Statement
