# Supplementary material for: Efficacy and Safety of Mifepristone in the Treatment of Male US Veterans With Posttraumatic Stress Disorder: A Phase 2a Randomized Clinical Trial
Source: JAMA Netw Open. 2023 May 9;6(5):e2310223. doi: 10.1001/jamanetworkopen.2023.10223 (PMC10170341; doi:10.1001/jamanetworkopen.2023.10223)
Supplement: Supplement 1. — eAppendix. CCTA #0004 Group Information eTable 1. Sensitivity Analyses of Clinical Response Rate (30% Reduction in CAPS Total Score) at 4 Weeks (Primary Endpoint) and 12 Weeks (Secondary Endpoint) eTable 2. Mifepristone Plasma and Metabolites at the End of 1-Week Dosing Phase by Clinical Responder Status (Limited to Mifepristone Group Only) eFigure 1. Plots of Estimated Means of Total CAPS Score Over Time eFigure 2. Plots of Cortisol and ACTH over Time by 4-Week Clinical Responder Status eTable 3. Institutional Review Boards [file jamanetwopen-e2310223-s001.pdf]

Supplementary Online Content

Golier JA, Li X, Bizien M, et al. Efficacy and safety of mifepristone in the treatment of male US veterans with posttraumatic stress disorder: a phase 2a randomized clinical trial. *JAMA Netw Open*. 2023;6(5):e2310223. doi:10.1001/jamanetworkopen.2023.10223

eAppendix. CCTA #0004 Group Information

eTable 1. Sensitivity Analyses of Clinical Response Rate (30% Reduction in CAPS Total Score) at 4 Weeks (Primary Endpoint) and 12 Weeks (Secondary Endpoint)

eTable 2. Mifepristone Plasma and Metabolites at the End of 1-Week Dosing Phase by Clinical Responder Status (Limited to Mifepristone Group Only)

eFigure 1. Plots of Estimated Means of Total CAPS Score Over Time

eFigure 2. Plots of Cortisol and ACTH over Time by 4-Week Clinical Responder Status

eTable 3. Institutional Review Boards

This supplementary material has been provided by the authors to give readers additional information about their work.

© 2023 Golier JA et al. *JAMA Network Open.*

eAppendix. CCTA #0004 Group Information

Planning Committee (responsibilities: study design): Julia Golier, MD; Dewleen Baker, PhD; Marcel Bizien, PharmD; Kush Kapur, PhD; Timothy Kimbrell, MD; Dan Lippe, MA; Julia Vertrees, Pharm.D; Rachel Yehuda, PhD; Domenic Reda, PhD. *Executive Committee (responsibilities: study conduct):* Julia Golier, MD; Robin Hurley, MD; Rachel Yehuda, PhD; Janine Flory, PhD; Domenic Reda, PhD; Xue Li, PhD; Dewleen Baker, PhD; Timothy Kimbrell, MD; Marcel Bizien, PharmD; Lisa Robin, MA, LPC; Brendan Bechard, BS. *Chairman's Office, Bronx Veteran Affairs Medical Center (VAMC), NY and Salisbury VAMC, NC (responsibilities: study administration and oversight):* Julia Golier, MD (Chair); Robin Hurley, MD (Co-Chair); Brendan Bechard, BS (national coordinator); Rachel Yehuda, PhD (PTSD consultant); Janine Flory, PhD (psychometrician consultant). *Clinical Science Research & Development Service, Office of Research and Development, Washington, DC (Responsibilities: Study Administration and Oversight):* Theresa Gleason, PhD (director); David Adjasoo, MS, MBA (senior program Analyst). *VA Cooperative Studies Program Coordinating Center (CSPCC), Hines, IL (responsibilities: study administration and oversight, data collection, verification and storage, statistical analysis)*: Domenic J. Reda, PhD (director); Xue Li, PhD (biostatistician); Lisa Robin, MA, LPC (project manager); Tom Sindowski, BS (project manager); Lizy Thottapurathu, MS (statistical programmer); Marie Reinhard, AA (data coordinator); Danuta Kowalczyk, BS (data management programmer); Tir Kelly, BA (data management programmer); Tom Koppes, RN (quality assurance specialist). *VA Cooperative Studies Program Clinical Research Pharmacy Coordinating Center (CSPCRPCC), Albuquerque, NM (responsibilities: study administration, oversight of rTMS Administration and Safety Monitoring)*: Marcel Bizien, PharmD (clinical research pharmacist); Donna Cain, BA (pharmaceutical project manager). *Participating Clinical Sites* (*responsibilities: recruitment and data collection*): *Bronx VAMC, NY:* Eran Chemerinski, MD (site investigator); Rachel Yehuda, PhD (co-investigator); Andreas Michaelides, PsyD (psychometrician); Samuel Krug, PhD (psychometrician); Kirklyn Escondo, BA (site coordinator); Emily Chapman, BA (site coordinator); Ahmed El-Razi, BA (site coordinator); Melissa Rothstein, BA (site coordinator). *Durham VAMC, NC:* Michael Hertzberg, MD (site investigator); Vickie Carpenter, MA (psychometrician); Hayley White, MA (site coordinator); Charles Treis, BA (site coordinator); Eric Berrios, AA (site coordinator). *San Diego VAMC, CA:* Dewleen Baker, PhD (site investigator, PTSD consultant); Kathryn Spaventa-Vancil, PhD (site coordinator); Heather Johnson, PhD (site coordinator); Michelene Vasil, MFT (site coordinator). *Albuquerque VAMC, NM:* Geraldo Villarreal, MD (site investigator); Ella Nye, PhD (psychometrician); Linnea Adamson, AA (site coordinator); Amy Mank, AA (site coordinator). *Salisbury VAMC, NC:* Robin Hurley, MD (site investigator); Calandra Hayes, MS (site coordinator); Mariah Delahanty, PharmD (site coordinator); Chenille Smith, MSW (site coordinator).

© 2023 Golier JA et al. *JAMA Network Open.*

eTable 1. Sensitivity Analyses of Clinical Response Rate (30% Reduction in CAPS Total Score) at 4 Weeks (Primary Endpoint) and 12 Weeks (Secondary Endpoint)

Analysis Methods		(n1, n2) [a]	Clinical Response Rate			Odds Ratio	
						Unadjusted	Adjusted [b]
			MIFE (%)	PBO (%)	Difference (95% CI)	OR (95% CI)	OR (95% CI)
	Baseline to 4-weeks follow-up (primary endpoint)						
CC		(39, 38)	38.5	31.6	6.9 (-14.4 to 28.1)	1.4 (0.5 to 3.5)	1.1 (0.4 to 3.0)
LACCF		(41, 39)	36.6	30.8	5.8 (-14.9 to 26.5)	1.3 (0.5 to 3.3)	1.0 (0.4 to 2.8)
WC		(41, 39)	36.6	30.8	5.8 (-14.9 to 26.5)	1.3 (0.5 to 3.3)	1.0 (0.4 to 2.8)
	Baseline to 12-week follow-up (secondary endpoint)						
CC		(32, 35)	31.3	40.0	-8.8 (-31.6 to 14.1)	0.7 (0.2 to 1.9)	0.6 (0.2 to 1.7)
LACCF		(41, 39)	36.6	35.9	0.7 (-20.4 to 21.8)	1.0 (0.4 to 2.6)	0.8 (0.3 to 2.2)
WC		(41, 39)	24.4	35.9	-11.5 (-31.5 to 8.5)	0.6 (0.2 to 1.5)	0.5 (0.2 to 1.3)

Abbreviation: OR stands for odds ratio; WC stands for Worst Cases where participants with missing CAPS total score at 4 weeks (12 weeks) were deemed as 4-week (12-week) clinical non-responders; LACCF stands for Last Available Case Carrying Forward where last available information on total CAPS score was used to impute the subsequently missed CAPS total score(s); and CC stands for complete cases at 4 weeks (12 weeks).

[a] n1 is the number of participants in mifepristone group, and n2 is the number of participants in placebo group.

[b] Adjusted for baseline PCL total score**.**

© 2023 Golier JA et al. *JAMA Network Open.*

eTable 2. Mifepristone Plasma and Metabolites at the end of 1-Week Dosing Phase by Clinical Responder Status (Limited to Mifepristone Group Only)

Measures	(n1, n2) [a]	Clinical Responders	Clinical Non-Responders [b]	Median Difference (95% CI) [c]	P-Value [d]
		Median (IQR)	Median (IQR)		
Plasma MIFE (ng/ml)	(14, 21)	1305.0 (980 to 1560)	1610.0 (1210 to 2140)	-368.0 (-850 to 90)	0.11
RU_42633 (ng/ml)	(14, 21)	1495.0 (1070 to 1860)	2100.0 (1340 to 2460)	-495.0 (-980 to 30)	0.06
RU_42698 (ng/ml)	(14, 21)	407.5 (265 to 654)	523.0 (335 to 739)	-112.5 (-315 to 76)	0.21
RU_42848 (ng/ml)	(14, 21)	1100.0 (815 to 1390)	1180.0 (955 to 1360)	-117.0 (-417 to 170)	0.30

[a] n1 is the number of participants in clinical responder group, and n2 is the number of patients in clinical non-responder group.

[b] Assume subjects who missed 4-week CAPS score are clinical non-responders. Results from Complete Case (CC) analysis that excluded 2 non-responders with missing 4-week CAPS score showed comparable results (p-value=0.11 for plasma MIFE, 0.06 for RU_42633, 0.24 for RU_42698, and 0.37 for RU_42848).

[c] Hodgest Lehmann estimate of median difference and 95% CI

[d] P-value is from Wilcoxon rank-sum test.

© 2023 Golier JA et al. *JAMA Network Open.*

eFigure 1. Plots of Estimated Means in Total CAPS Score Over Time

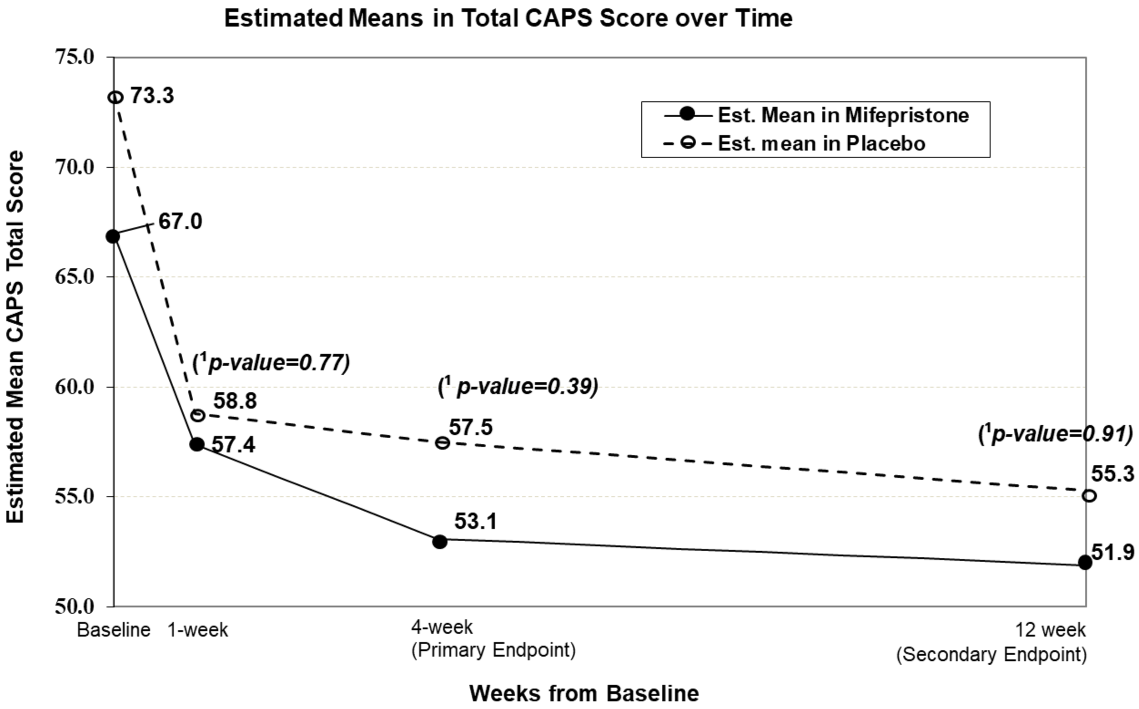

[1] p-values comparing the estimated mean scores between treatments at weeks 1, 4 and 12. The p-values were from the longitudinal repeated measures Covariance-pattern model (baseline CAPS score are part of outcomes) by specifying appropriate contrasts for the model. The p-value for time effect is <0.0001 and the p-value for treatment x time interaction is 0.42.

Additional analysis in change scores adjusted for baseline CAPS total score as a covariate showed similar results: mean changes of CAPS score from baseline to week 1 [-9.76 in MIFE vs. -14.31 in PBO; difference = 4.55 (95% CI: -1.91 to 11.00, p-value = 0.16]; from baseline to week 4 [-14.04 in MIFE vs. -15.52 in PBO; change score difference =1.48 (95% CI: -6.62 to 9.59), p-value = 0.72], and from baseline to week 12 [-15.05 in MIFE vs. -17.94 in PBO; change score difference = 2.89 (95% CI: -7.26 to 13.03), p-value = 0.57].

© 2023 Golier JA et al. *JAMA Network Open.*

eFigure 2. Plots of Cortisol and ACTH over Time by 4-Week Clinical Responder Status

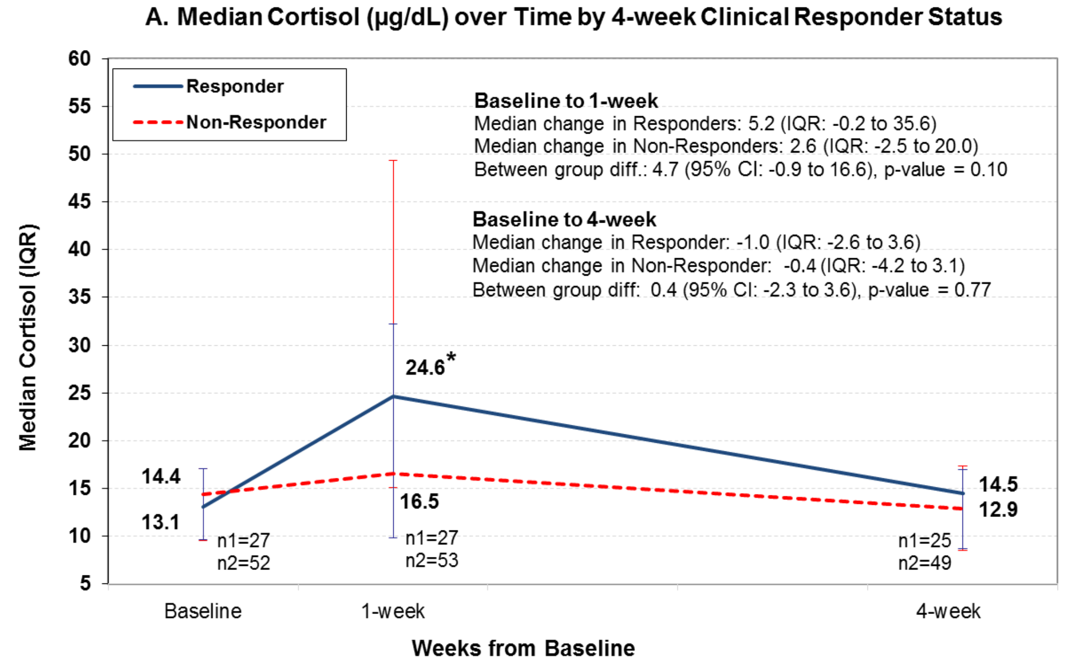

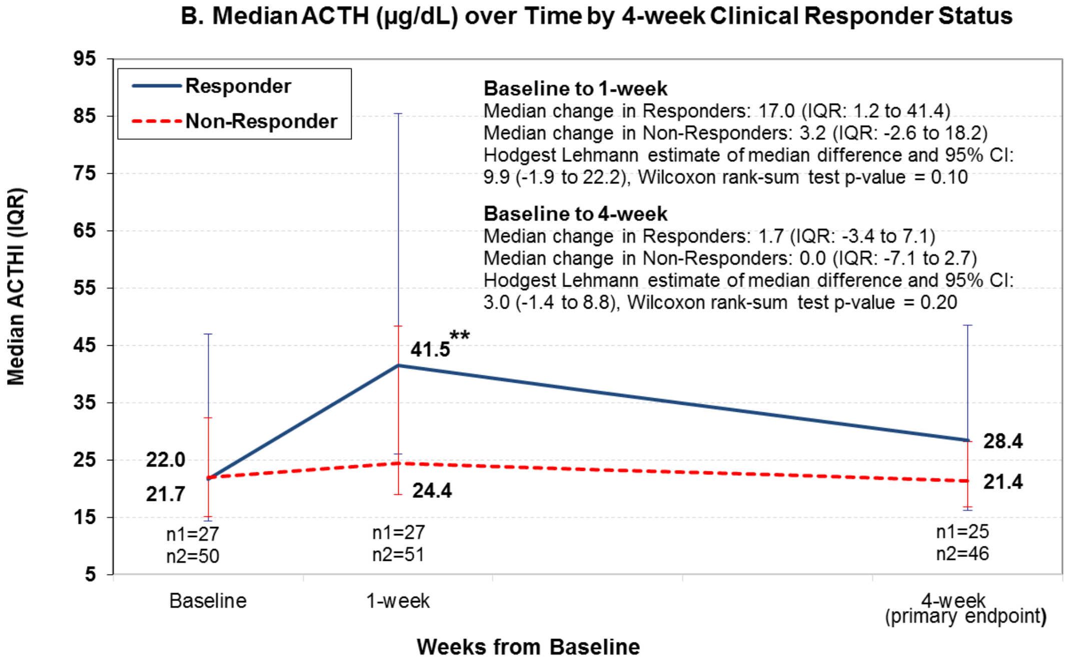

n1 and n2 are, respectively, the number of participants in the responder group and the non-responder group. Two outliers (one in each treatment group) with extremely large ACTH values were excluded from ACTH analysis. Subjects who missed 4-week CAPS score are defined as clinical non-responders. Bars around the median are the IQR (1st quartile, 3rd quartile). Single asterisk indicates 0.05 < *p*-value (Wilcoxon rank-sum test) < 0.10 and double asterisks for *p*-value < 0.05.

© 2023 Golier JA et al. *JAMA Network Open.*

eTable 3. Institutional Review Boards

**Role in the Study**	**Location**	**Institutional Review Board (IRB) Name**
Chair's Office	Bronx VAMC, New York, NY	Institutional Review Board Bronx VAMC Research
Recruiting Site	Bronx VAMC, New York, NY	Institutional Review Board Bronx VAMC Research
Recruiting Site	Durham VAMC, Durham, NC	Institutional Review Board Durham VAMC Research
Recruiting Site	San Diego VAMC, San Diego, CA	Institutional Review Board San Diego VAMC Research
Recruiting Site	Albuquerque VAMC, Albuquerque, NM	Institutional Review Board Albuquerque VAMC Research
Recruiting Site	Salisbury VAMC, Salisbury, NC	Institutional Review Board Salisbury VAMC Research
Cooperative Studies Program Coordinating Center (CSPCC)	Edward Hines, Jr. VA Hospital Hines, IL	Edward Hines, Jr. VA Hospital IRB

*VAMC: Veteran Affairs Medical Center

© 2023 Golier JA et al. *JAMA Network Open.*
