# Supplementary material for: Efficacy and Safety of Mifepristone in the Treatment of Male US Veterans With Posttraumatic Stress Disorder: A Phase 2a Randomized Clinical Trial
Source: JAMA Netw Open. 2023 May 9;6(5):e2310223. doi: 10.1001/jamanetworkopen.2023.10223 (PMC10170341; doi:10.1001/jamanetworkopen.2023.10223)
Supplement: Supplement 2. — Trial Protocol [file jamanetwopen-e2310223-s002.pdf]

STUDY PROTOCOL: COOPERATIVE CLINICAL TRIAL AWARD PROGRAM #0004:

Novel Therapeutics in PTSD: A Randomized Clinical Trial of Mifepristone

1. Rationale

1.a. Statement of the Problem

Post-traumatic stress disorder (PTSD) is a common psychiatric disorder that can be precipitated by severe psychological trauma including combat, accidents, and physical or sexual assault. Left untreated, PTSD can become a chronic, disabling condition associated with significant depression, aggression, family disruption, and substance abuse (Bremner et al., 1996). There is accumulating evidence that PTSD is also associated with increased morbidity and mortality from cardiovascular disease, suicide, and other illnesses (Bell and Nye, 2007; Boscarino, 2008; Jakupcak et al., 2009). Given the high cost of this disorder to veterans, their families, and society at large, improving its treatment is a high priority within the Department of Veterans Affairs (VA).

In a VA-commissioned review of the state of treatment of PTSD, the Institute of Medicine (IOM) did not find evidence to support the efficacy of commonly used psychotropic medications (IOM, 2008). This is of particular concern since pharmacotherapy is a major treatment modality for PTSD in the VA. Among veterans who initiate PTSD treatment at the VA, 50% are prescribed psychotropic medication within the first year (Spoont et al., 2010); among veterans in chronic care for PTSD, 80% are receiving psychotropic medications (Mohamed and Rosenheck, 2008). The vast majority are prescribed an antidepressant, primarily selective serotonin reuptake inhibitors (SSRIs). However, controlled trials have not found SSRIs to be effective in PTSD (Hertzberg et al., 2000; Friedman et al., 2007). In a 12 week placebo-controlled trial of sertraline in veterans, active treatment was not associated with improvements in PTSD, its associated symptoms (depression, anxiety, sleep disturbance), or quality of life (Friedman et al., 2007). Antipsychotics, mood-stabilizers, sedatives, and hypnotics are also widely used, alone and in combinations, despite limited efficacy data and some concerns regarding safety (e.g., metabolic and neurologic side effects). The extensive use of polypharmacy, incorporating off-label medications and medications that have not been shown to be effective, highlights the limitations in the current psychopharmacological approach to PTSD. There is clearly a need to develop more effective pharmacological treatments. Whereas the medications that are widely used to treat PTSD were developed for use in other disorders and are prescribed for long-term use, medication strategies that specifically target PTSD symptoms and/or pathophysiology may be more effective and possibly even curative. Therefore, we propose to examine whether targeting hypothalamic-pituitary-adrenal (HPA) axis dysregulation in PTSD with mifepristone – which recalibrates the HPA axis through peripheral and central mechanisms – will be of therapeutic benefit.

Decades of work by VA investigators and others have demonstrated that dysregulation of the HPA axis is a feature of PTSD pathophysiology and an important potential biological treatment target. The neuroendocrine profile in PTSD is a unique one, best characterized by increased levels of corticotrophin-releasing hormone (CRH) (Bremner et al., 1997; Baker et al., 1999) and enhanced sensitivity to exogenous glucocorticoids in some, but not all, target tissue in the absence of hypercortisolism. Indeed, despite the activation of CRH pathways, 24 hour urinary and plasma cortisol levels are often found to be low in PTSD (Kanter et al., 2001; Yehuda, 2002). Numerous studies in veterans and civilians have demonstrated enhanced suppression of cortisol by the synthetic glucocorticoid dexamethasone (DEX) in PTSD (e.g., Yehuda et al., 1993; Yehuda et al., 2002; Newport et al., 2004), which appears to reflect enhanced glucocorticoid receptor (GR) sensitivity at the level of the pituitary. A prospective study examining GR number in lymphoyctes before deployment found that a greater number of GR predicted PTSD to combat trauma, thus providing support for the view that GR alterations are risk factors for PTSD (van Zuiden et al., 2011). What is unknown is whether the observed GR alterations in multiple target tissues represent a primary alteration or a secondary adaptation to disturbances in other aspects of HPA axis activity.

One hypothesis is that the up-regulation of glucocorticoid receptors in PTSD and increased sensitivity to dexamethasone are secondary adaptations attempting to normalize inadequate glucocorticoid signaling (Raison and Miller, 2003). Low cortisol has been described in the initial aftermath to trauma in some trauma survivors who go on to develop PTSD and is often observed in those with chronic PTSD (Yehuda 2002). An inadequate cortisol response at the time of trauma or in its aftermath may lead to a failure to constrain stress

responsive systems following stress exposure leading to the cascade of neurobiological events associated with PTSD. Since low cortisol is not an invariable feature of the disorder it is important to consider that inadequate cortisol signaling in PTSD can also be inferred by consideration of the target tissues that are regulated by glucocorticoids (Raison and Miler et al., 2003). Glucocorticoids are known to mobilize immune responses during stress, restrain the sympathetic nervous system and inhibit CRH activity in the hypothalamus. In PTSD there is consistent evidence of enhanced sympathetic nervous system (SNS) leading to increased catecholamine levels (Kosten et al., 1987; Southwick et al., 1997), increased activity of CRH pathways (Bremner et al., 1997; Baker et al., 1999), immune activation and a shift toward a pro-inflammatory cytokine profile (Raison and Miller, 2003; von Känel et al., 2007). These neurobiological markers have been consistently described in PTSD could be explained by inadequate glucocorticoid signaling. These neurobiological makers are believed to contribute to the behavioral, metabolic, and immune alterations associated with this disorder (Raison and Miller, 2003). Thus, it is important to determine whether recalibrating a dysregulated HPA axis and enhancing glucocorticoid signaling pathways is of therapeutic benefit in PTSD.

Mifepristone is of particular interest for development in PTSD because it is a glucocorticoid receptor antagonist that is hypothesized to recalibrate the HPA axis through blockade of peripheral and central GR and enhance central glucocorticoid signaling (Belanoff et al., 2002; Karssen et al., 2003; Buckley et al., 2008). Peripheral GR blockade results in increased cortisol and adrenocorticotropin hormone (ACTH) levels owing to blockade of cortisol's feedback inhibition of the HPA axis. At high doses mifepristone also has central effects. It crosses the blood-brain barrier, as evidenced by animal studies that demonstrate that there are comparable levels of drug in both plasma and brain after a peripheral injection (Karssen et al., 2003), although it is rapidly cleared. Mifepristone increases the uptake of cortisol into the brain as a result of its effect on the glycogen pump at the blood-brain barrier. Under normal circumstances the glycogen pump hampers the uptake of cortisol; however, mifepristone and its metabolites inhibit the efflux of cortisol via this pump which facilitates the uptake of cortisol into the brain (Sarkar, 2002). Cortisol exerts its effects through two types of receptors in the brain: glucocorticoid receptors and mineralocorticoid receptors (MR). The balance of MR/GR-mediated actions is critical to homeostatic processes in the brain; an imbalance is associated with HPA axis dysregulation (De Kloet and Derijk, 2004). The marked increase in cortisol in the brain following mifepristone administration, together with GR blockade, leads to increased activation of MRs (De Kloet et al., 1998). The attendant shift in balance of MR/GR occupancy is hypothesized to recalibrate the HPA axis by normalizing hormonal set points (De Kloet et al., 1998; Belanoff et al., 2002; Buckley et al., 2008). In PTSD, enhanced central glucocorticoid signaling and normalization of HPA axis regulation could constrain stress responsive systems that are disrupted in PTSD leading to clinical improvement.

To date there are no published studies supporting the use of mifepristone in PTSD, but there is powerful evidence that glucocorticoid manipulation can have therapeutic effects in traumatic stress (Yehuda, 2009). In a study of the treatment of septic shock, it was discovered that ICU patients who received high doses of glucocorticoid were less likely to develop PTSD than those who had not; subsequent randomized controlled trials have confirmed that high dose steroids prevent the development of PTSD in critically ill and surgical patients (Schelling et al., 2004; Schelling et al., 2006). In these patients, high doses of hydrocortisone are hypothesized to work by enhancing glucocorticoid signaling impaired by critical illness. The apparent mechanisms involve down regulation of the stress response, inhibition of traumatic memory retrieval, and facilitation of extinction of aversive information (Schelling et al., 2006). In a small pilot crossover study of low-dose cortisol in PTSD, cortisol was associated with significant reduction in PTSD symptoms (Aerni et al., 2004). The idea of using steroids for PTSD prophylaxis is an intriguing one, but we do not propose hormone replacement as a treatment for chronic PTSD owing to the established adverse effect profile of glucocorticoids when administered at high doses or over long periods of time and because hormone replacement at low doses does not serve to recalibrate the HPA axis in the way that mifepristone is hypothesized to do.

There are data that suggest short-term administration of high doses of mifepristone has beneficial clinical and neuroendocrine effects in other neuropsychiatric conditions which persist beyond the period of medication discontinuation (Belanoff et al., 2002; Young et al., 2004; Gallagher et al., 2005; DeBattista et al., 2006). We also have striking pilot data in a small number of veterans with PTSD that suggests that mifepristone treatment may be efficacious; as such, further study into whether and how this drug can be developed for use in PTSD is warranted. In the absence of published data in PTSD and in the presence of

abundant safety data in other conditions, an exploratory trial for efficacy is the next logical step in developing this drug for use in veterans with PTSD. This study is designed for that purpose.

1.b. Overview

A multicenter-center Phase IIa, double-blind, randomized controlled trial of a 600 mg dose of mifepristone and placebo is being proposed to determine whether there is sufficient evidence of a signal for short-term (one and three months) efficacy in veterans with chronic PTSD to warrant a Phase III trial. For this, 90 veterans with PTSD will be randomized to either mifepristone or placebo. To determine whether there is a signal, we will use statistical selection theory (see Simon et al., 1985). The final outcome will compare the proportion of clinical responders one month after treatment under the scenario: mifepristone 600 mg/day for 7 days vs. placebo. A clear benefit of this method is that it requires a much smaller sample size than traditional hypothesis testing, resulting in a reduction of both clinical costs and adverse events; this approach is widely used for Phase II drug development and testing in the pharmaceutical industry to determine whether to pursue further clinical testing of a specific compound. Based on the outcome of this exploratory trial, we will make a determination regarding whether to pursue a larger and more definitive Phase III study of mifepristone.

The innovation of studying mifepristone is twofold. The first is the application of this glucocorticoid receptor antagonist to the treatment of PTSD, which has not been studied before. The second is the use of a short-term dosing strategy that is novel in the context of classic pharmacological treatment of mental disorders. Whereas most pharmacological treatments are recommended for long-term use, the available data suggest that short-term use of a high dose of mifepristone can have enduring effects through a re-setting of the HPA axis. Short-term or intermittent dosing strategies may be particularly appealing to veterans, as complaints regarding side effects associated with long-term pharmacotherapy are common. If the initial trials in PTSD are successful, subsequent studies could assess myriad potential clinical applications in PTSD including single or intermittent dosing as monotherapy, as an adjunctive treatment to enhance or accelerate the effects of other pharmacological treatments or psychotherapy, and for PTSD prophylaxis following traumatic stress.

The CCTA mechanism is being pursued since the ultimate goal is to develop a pharmacological treatment strategy that is efficacious in veterans with PTSD. The use of this mechanism will ensure that adequate clinical, scientific, and pharmaceutical oversight and resources will be available to pursue this goal; Phase II trials fall within the purview of the CCTA mechanism.

In this initial trial we propose to study the effects of mifepristone in veterans with PTSD in order to better detect a signal if there is one. Consistent with previous studies in neuropsychiatric disorders (Belanoff et al., 2002; Young et al., 2004; Gallagher et al., 2005; DeBattista et al., 2006; Gallagher et al., 2008; Blasey et al., 2009), participants will be treated daily for one week with a high dose of mifepristone (600 mg/day) or placebo and clinical outcomes will be determined at one month. The primary outcome will be the clinical responder rate, with responders defined as a 30% reduction in the total PTSD symptom severity score (past week symptom status) from baseline to four weeks derived from the Clinician Administered PTSD Scale (CAPS). This definition of responder status has been used consistently in PTSD clinical trials. The response will be based on the change in CAPS (past week symptom status) score from baseline to four weeks. To evaluate the durability of the effects of this short-term treatment, participants will be assessed for up to three months.

1.c. <u>Specific Objectives</u>

1.c.1. *Primary Objective*

1.c.1.a.To determine whether 600 mg of mifepristone daily for one week in male veterans with chronic PTSD yields a sufficiently high proportion of clinical responders after one month to warrant more extensive and definitive research.

1.c.2. *Secondary Objectives*

1.c.2.a. To study the trajectories of CAPS (past week symptom status) scores over the study duration for mifepristone (600 mg/day) and placebo.

1.c.2.c. To determine if a Phase III study is justified.

1.c.2.d. To compare study drug related adverse events (AEs) and serious adverse events (SAEs) in the two groups.

1.d. <u>Outcome Measures</u>

1.d.1. *Primary Outcome*

The primary clinical outcome measure will be the presence or absence of a clinical response, defined as a 30% or greater reduction in total CAPS (past week symptom status) score from baseline to four weeks.

1.d.2. *Secondary Outcomes*

The secondary outcome measures will be the changes in CAPS (past week symptom status) score from baseline to weeks 1, 4, and 12 to capture time by treatment interaction, the proportion of clinical responders at 12 weeks for deciding sustainability, and the percentage of study drug related AEs and SAEs to study safety and tolerability of mifepristone.

1.d.3. *Descriptive Outcomes*

The impact of mifepristone on other clinical parameters will be described, including the three PTSD symptom sub-scales from the CAPS (intrusion/re-experiencing, avoidance/emotional numbing, and hyperarousal), depression (Beck Depression Inventory (BDI)), PTSD (PTSD Checklist (PCL)), sleep quality (Pittsburgh Sleep Quality Index (PSQI)), anger (State-Trait Anger Expression Inventory (STAXI)) and functional impairment (functional impairment measure (item 24) on the CAPS). The neuroendocrine effect of mifepristone in PTSD will be assessed by measuring the changes in plasma cortisol and ACTH levels from baseline to immediately after one week of treatment and from baseline to four weeks. The relationship of plasma cortisol and ACTH levels to clinical response will also be explored. As an additional descriptive measure, plasma levels of mifepristone and its metabolites will be assessed immediately after one week of treatment, and its relationship to clinical response and the trajectories of CAPS scores will be assessed.

2. Background and Significance

2.a. <u>Background</u>

2.a.1. *Clinical and neuroendocrine profile of mifepristone*

Animal and human studies have shown that mifepristone is a potent glucocorticoid receptor antagonist that blocks the effects of endogenous and exogenous glucocorticoids (i.e. cortisone, cortisol, and dexamethasone) and has peripheral and central effects (Bertagna et al., 1984; Gaillard et al., 1984; Heikinheimo et al., 2003). Cortisol levels increase in response to acute HPA axis activation and then inhibit their further release through negative feedback inhibition of the HPA axis, as depicted in Figure1. Accordingly, the acute blockade of GR by mifepristone and its metabolites which interrupts this negative feedback loop, results in a compensatory increase in cortisol and ACTH levels (Bertagna et al., 1984; Raux-Demay et al., 1990). While ambient cortisol levels increase with mifepristone, cellular assays reveal that mifepristone decreases peripheral glucocorticoid bioavailability through blockade of GR (Heikinheimo et al., 2003). At high doses mifepristone crosses the blood-brain barrier, as evidenced by animal studies that demonstrate that there are comparable levels of drug in both plasma and brain (Sarker et al., 2002; Karssen et al., 2003). Mifepristone and its active metabolites inhibit the efflux of cortisol via the glycogen pump at the blood-brain barrier which facilitates the uptake of cortisol into the brain (Sarkar, 2002); brain levels are further increased due to the increased peripheral levels.

Figure 1.

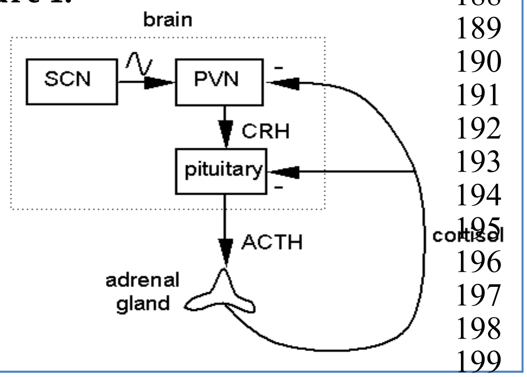

Mineralocorticoid receptors (MR), which are activated at lower doses of cortisol, help to maintain neuronal homeostasis and limit the disturbance caused by acute stress whereas GRs, which are occupied at higher levels of cortisol, help to facilitate recovery from a stressor. The balance of MR/GR-mediated actions is critical to homeostatic processes in the brain, and an imbalance leads to HPA axis dysregulation and associated aberrations in metabolism, immune function, and memory function (De Kloet and Derijk, 2004). The attendant shift in balance of MR/GR occupancy is hypothesized to recalibrate the HPA axis by normalizing

hormonal set points (Belanoff et al., 2002; Buckley et al., 2008) which could reverse some of the stress-related neurobiological and allostatic changes that are associated with this disorder. Since the HPA axis in PTSD appears to be dysregulated in a unique way, the full range of effects of GR antagonism in PTSD cannot be fully predicted based on animal studies or studies in major depression. A systematic, empiric approach is needed to examine the potential range of therapeutic effects in PTSD and their neuroendocrine correlates.

Mifepristone has been studied in psychotic major depression (PMD). PMD and PTSD are both characterized by elevated levels of CRH but many of the other neuroendocrine alterations are in opposite directions in these two disorders. Thus, efficacy or lack of it would not predict efficacy in the other disorder. The clinical trial data are summarized to highlight the existence of evidence for the central effects of mifepristone and recalibration of the HPA axis as well as abundant safety data (see section 2.a.3.).

The initial trials of mifepristone in PMD include: 1) a crossover trial in which mifepristone (600 mg/day x 4 days) was associated with rapid improvement in depression (Belanoff et al., 2001); 2)an open-label trial in which high dose mifepristone (600 mg/day or 1200 mg/day) for one week had substantial improvement in depression and psychosis compared to a low-dose mifepristone (50 mg/day) (Belanoff et al., 2002); and 3) a double-blind trial in which 7 of 15 in the mifepristone group showed a 50% reduction in psychotic symptoms compared to only 2 of 15 in the placebo group (Flores et al., 2006). The first Phase III trial was positive; in a double-blind trial of 221 patients with psychotic major depression, persons in the mifepristone group were significantly more likely to achieve a clinical response than those in the placebo group (DeBattista et al., 2006). In a subsequent Phase III trial of one week of mifepristone (600 mg/day), there was a site-by-treatment interaction (Blasey et al., 2009). In the initial 20 study sites involved from the trial outset, there was a significantly greater proportion of responders at day 56 in the mifepristone than the placebo group (26% vs. 13%). In contrast, in the 9 additional sites added to boost enrollment, there was a higher placebo response rate. An analysis of trough mifepristone levels, which was specified *a priori*, showed that the response rates were significantly higher in patients whose plasma mifepristone levels were at or above the pre-specified threshold level (1800 ng/ml) compared to placebo; the effect was evident both when examining the original sites in the study (active response vs. placebo response of 46% vs. 17%) and when all sites were included (41% vs. 23%) (Blasey et al., 2009).

The data demonstrate that mifepristone can induce beneficial clinical effects that persist beyond the period of active treatment (Belanoff et al., 2002; Young et al., 2004). It has been hypothesized that enduring effects of mifepristone may result from a recalibration of the HPA axis. One proposed mechanism is that the acute blockade of GR, increase in cortisol levels, and binding of cortisol to the MR (which are not blocked by mifepristone) would lead to the down-regulation of these receptors and a resetting of the HPA axis. Such effects are hypothesized to be enduring clinical effects that have been observed after drug discontinuation (Belanoff et al., 2002; Flores et al., 2006). Supporting this hypothesis, a recent analysis found that cortisol levels in patients with bipolar disorder and schizophrenia increased acutely following treatment with mifepristone (600mg/day x 7 days) compared to placebo; at day 21, cortisol levels had declined from baseline in the mifepristone group, suggesting normalization of the HPA axis (Gallagher et al., 2008). These data provide preliminary evidence that mifepristone can recalibrate the HPA axis and induce enduring therapeutic change in a way that may be relevant in the treatment of PTSD.

2.a.2.*Rationale for trial of mifepristone in veterans with PTSD*

2.a.2.a. Mifepristone and the neuroendocrinology of PTSD

Among the most replicated neuroendocrine findings in PTSD (the majority of which have been demonstrated in veterans) have been of elevated levels of corticotropin-releasing factor (CRF) in the cerebrospinal fluid (CSF) (Bremner et al., 1997; Baker et al., 2005) and an exaggerated suppression of cortisol to a low dose of the synthetic glucocorticoid dexamethasone (DEX). The finding of enhanced suppression of cortisol to low-dose DEX was first observed in Vietnam veterans with PTSD (Yehuda et al., 1993). With a few exceptions, greater suppression of cortisol to low-dose DEX has consistently been found in PTSD participants compared to unexposed and/or trauma-exposed controls without PTSD; the finding has also been demonstrated in samples of persons exposed to combat, natural disasters, domestic violence, the Holocaust, and childhood physical and sexual abuse (Newport et al., 2004; Griffin et al., 2005). More recent studies have demonstrated increased suppression of ACTH to DEX (Yehuda et al., 2004; Newport et al., 2004), confirming

increased glucocorticoid responsivity at the level of the pituitary. Enhanced negative feedback inhibition of the HPA axis in PTSD could help to explain why 24-hour basal cortisol levels are not typically elevated, and indeed are even sometimes low (reviewed in Yehuda, 2002), despite evidence of central HPA axis activation and exaggerated HPA axis responsivity (Rasmusson et al., 2001; Bremner et al., 2003). It is presumed that the enhanced sensitivity to glucocorticoids reflects greater responsiveness of glucocorticoid receptors (GR), as suggested by changes in GR number and response following challenge with DEX (Yehuda et al., 2003). That enhanced GR responsiveness is a risk factor for PTSD has recently been confirmed in a large prospective study of deployment-related PTSD. Basal cortisol level was not a predictor of PTSD, but the number of lymphocyte glucocorticoid receptors was significantly higher in participants who developed PTSD symptoms following deployment than those who did not (van Zuiden et al., 2011). It has been hypothesized that enhanced GR responsiveness is a secondary adaptation to decreased hormone bioavailability (Raison and Miller, 2003), which is suggested by the finding of lower cortisol in some studies in PTSD (Yehuda, 2002). Since mifepristone is a glucocorticoid receptor antagonist and increases brain cortisol it could be beneficial both with respect to enhanced GR sensitivity and inadequate signaling. Inadequate glucocorticoid signaling, whether as a result of low cortisol or other alterations, would set the stage for an inadequate response to acute stress and a failure to constrain stress responsive systems (reviewed in Yehuda, 2009). Inadequate glucocorticoid signaling would in turn release the sympathetic nervous system (SNS) and CRH from inhibitory control, leading to increased sympathetic catecholamine levels (Raison and Miller, 2003), CRH levels (Bremner et al., 1997; Baker et al., 1999), and immune activation (Raison and Miller, 2003), which have been described in PTSD. Inadequate glucocorticoid signaling in the aftermath of trauma may result in a cascade in which there is increased SNS activation, leading to an exaggerated catecholamine response to the trauma, which in turn could initiate a process in which traumatic memories become “over-consolidated” or inappropriately remembered due to an exaggerated level of distress and arousal (Pitman, 1989). Failure to properly contain the SNS response to traumatic reminders could perpetuate the intrusive and hyperarousal symptoms of chronic PTSD, leading to the elaboration of avoidance symptoms that commonly occur in the disorder over years or even decades (Yehuda, 2009).

2.a.2.b. Potential mechanisms by which mifepristone could impact PTSD-associated alterations in brain function

Since GR and MR are expressed in key brain regions implicated in PTSD, normalization of HPA axis regulation could lead to symptomatic and cognitive improvement across multiple domains. Convergent structural and functional neuroimaging studies and neuropsychological studies support a model of altered neurocircuitry in PTSD best characterized by exaggerated amygdala responses and deficient prefrontal cortical (PFC) and hippocampal function (reviewed in Rauch et al., 2006). These brain regions express GR and MR and are regulated by glucocorticoids. It is not yet known to what extent impairments in the functioning of these brain regions or alterations in their circuitry are a function of aberrant neuroendocrine activity, nor to what extent these impairments contribute to the HPA axis alterations in PTSD. However, studies have demonstrated differential effects of exogenous glucocorticoids on declarative memory and working memory in PTSD, suggesting altered central sensitivity to glucocorticoids in PTSD, which may be mediated through effects on the hippocampus and amygdala (Bremner et al., 2004).

There is abundant evidence of impaired hippocampal function in PTSD, as suggested by impairments in explicit/declarative memory and paired associate learning (Gilbertson et al., 2001; Golier et al., 2003; Bremner et al., 2004). Imaging studies provide evidence of reduced hippocampal activation in response to cognitive activation techniques (Bremner et al., 2003), smaller hippocampal volumes (Gurvits et al., 1996; Stein et al., 1997), and decreased hippocampal N-acetyl aspartate (indicative of impaired neuronal integrity), which was positively associated with cortisol level in the absence of hypercortisolemia (Neylan et al., 2003). Although some of these hippocampal and HPA axis characteristics may reflect pre-morbid risk for PTSD (Gilbertson et al., 2002), they do not appear to be immutable. Indeed SSRI treatment of PTSD in civilians is accompanied by an increase in hippocampal volume, improved declarative memory performance, and reduced cortisol reactivity to stress (Vermetten et al., 2006). Thus, if there is altered glucocorticoid regulation of the hippocampus, GR blockade and the subsequent up-regulation of hippocampal MR could lead to improvement in PTSD-related symptoms and memory impairments. In animal studies, mifepristone reverses glucocorticoid-

mediated impairments in hippocampal morphology and function. For example, in rats, it protects against glucocorticoid-induced cell death in the hippocampus, fully reverses the deleterious effects of chronic stress on hippocampal synapses, and improves spatial memory, a hippocampal-dependent form of memory, when administered continuously (Oitzl et al., 1998).

Deficient activity of the medial PFC in response to aversive stimuli (Shin et al., 2005) has been observed in PTSD, and symptom improvement is associated with increased medial PFC activity (Seedat et al., 2004). PTSD is also associated with deficits in working memory (Vasterling et al., 1998; Stein et al., 2002), a memory process mediated in part by the PFC. In healthy adults, working memory is very sensitive to the effects of glucocorticoids (Lupien et al., 1999; Young et al., 1999), and working memory in PTSD participants is more sensitive to the effects of hydrocortisone than in healthy controls; this finding suggests the possibility of greater glucocorticoid sensitivity in this region. Altered activity of the amygdala in PTSD, evidenced by increased amygdala activity in response to traumatic reminders and aversive stimuli (Liberzon et al., 1999; Shin et al., 2005) has also been shown. Animal studies describe a complex interplay between the amygdala and the HPA axis, and their effects on memory. Glucocorticoids increase amygdala activation; amygdala activation can activate the HPA axis and the noradrenergic system which, through secretion of norepinephrine, can stimulate CRF release and further enhance cortisol secretion in a feed-forward system. In broad outline, such studies suggest that normaliziation of HPA axis activity would impact amygdalar activity, perhaps improving the associated anxiety and anger common in PTSD.

These human and animal studies demonstrate that there are multiple brain sites implicated in PTSD that have reciprocal interactions with the HPA axis, the normalization of which could impact PTSD-related symptoms, cognitive impairments, and HPA axis alterations. The full range of acute and chronic effects of GR antagonism in PTSD cannot be predicted based on either animal studies or studies in other human disease states since treatment with mifepristone would modulate glucocorticoid activity in multiple interconnected brain regions simultaneously in systems that are dysregulated, in a unique way, at baseline. Thus, a systematic, multidisciplinary approach is needed to examine the full range of clinical and neuropsychological effects of mifepristone in PTSD and their relationship to neuroendocrine effects.

2.a.3. *Safety, tolerability and dosing*

The proposed study is grounded in the use of established clinical and experimental methods. In all previous studies of mifepristone in neuropsychiatric disorders, a pulse dose strategy has been employed with administration of mifepristone for 4-8 days. The majority of these studies used a dose of 600 mg/day (Belanoff et al., 2001; Belanoff et al., 2002; Young et al., 2004; Simpson et al., 2005; Gallagher et al., 2005; Flores et al., 2006; DeBattista et al., 2006; Blasey et al., 2009); 1200 mg/day has been studied in psychotic depression (Belanoff et al., 2002) and is being used in current studies of mifepristone in psychotic depression. In a recent Phase III trial of 600 mg/day of mifepristone in psychotic depression, a dose-response relationship between plasma concentration of mifepristone and treatment response was found. Only about one-third of the participants achieved what was previously determined to be the optimal blood level of mifepristone in depression. If a similar dose-response relationship exists in PTSD, some participants would need a higher dose of mifepristone to achieve clinical response; yet, in our pilot data there is preliminary evidence of a treatment effect at 600 mg/day. Accordingly, we will examine the effects of 600 mg/day of mifepristone and post-treatment mifepristone levels on clinical response. According to Dr. Roe, rash is estimated to occur in 1% of persons treated with 300 mg/day of mifepristone, 3.3% of persons treated at 600 mg/day, and 5.5% of persons treated with 1200 mg/day (Corcept Therapeutics).

With respect to HPA axis activity, based on the results of the ACTH and CRF stimulation tests, pituitary and adrenal reserves are maintained during mifepristone treatment (Bertagna et al., 1994). Chronic treatment with mifepristone does not appear to be associated with the development of iatrogenic adrenal or pituitary insufficiency. Based on published reports and personal communication with Dr. Robert Roe of Corcept Therapeutics, adrenal insufficiency has never been observed with one week of mifepristone treatment in depressed patients. (See also the section on monitoring adverse events, 4.i., for details on safety monitoring.) Nonetheless, since mifepristone is contraindicated in adrenal insufficiency, for safety reasons participants with a known history of this disorder or with low screening plasma cortisol levels will be excluded.

In a study of 7-14 days of treatment with mifepristone (10 mg/kg/day) (Laue et al., 1990), no changes

were observed in multiple laboratory measures including complete blood cell count, erythrocyte sedimentation rate, C-reactive protein, antinuclear antibodies (ANA), rheumatoid factor, and multiple quantitative immunoglobulin measures. In multiple subsequent trials of mifepristone, the drug was not associated with significant side effects (Belanoff et al., 2001; Belanoff et al., 2002; Young et al., 2004; Simpson et al., 2005; DeBattista et al., 2006; Flores et al., 2006). In the largest of these trials, psychotically depressed individuals were treated with 600 mg/day of mifepristone (n=105) or placebo (n=116) for one week, followed by usual treatment; there were no significant differences in the incidence of rash or multiple other potential side effects including headache, nausea, vomiting, constipation, dizziness, insomnia, sedation, abdominal pain, or toothache (DeBattista et al., 2006).

Another issue concerns whether it is ethical and safe to enroll veterans in a placebo-controlled medication trial. We believe it is ethically justified in the present trial since FDA-approved pharmacological options for the treatment of PTSD have not been shown to be effective in veterans with PTSD (Hertzberg et al., 2000; Friedman et al., 2007), and since the IOM report concluded that there is no evidence of efficacy for commonly used pharmacotherapies in veterans with PTSD For safety purposes, many safeguards have been put in place including the exclusion of patients at risk for suicide, frequent monitoring of suicidality and clinical state throughout the project, and the ability of the investigator to adjust medications or treatment plan if needed, without dropping the patient from the study, if his clinical condition deteriorates; these safeguards are described in detail elsewhere (see section 4.i. for details on safety monitoring). Numerous steps have been taken to ensure that patients will be safe and will receive standard pharmacotherapy if necessary. Overall, the proposed study is designed with a favorable risk to benefit ratio for male veterans with PTSD.

As per the medication package insert veterans will be told to swallow the tablets whole. The tablets are quite hard and film coated and therefore difficult to break. Unused tablets will be returned to the Albuquerque CSPCRPCC. Since this drug can cause loss of pregnancy in women, the medication should be kept away from pregnant women and women who could become pregnant.

2.b. Significance

It is estimated that 30% of men and 15% of women who served in the Vietnam War developed PTSD (Kulka et al., 1990), and approximately 21% of OIF/OEF veterans seen at the VA from 2002 to 2008 have been diagnosed with PTSD (Seal et al., 2009).Accordingly, there is an urgent need to design treatments that are more effective for this growing population. The significance of this proposal is that it tests a pharmacological approach in PTSD that is innovative in several ways. Whereas most compounds tested for efficacy in PTSD are psychoactive medications developed for other clinical indications and taken daily, this approach seeks to translate clinical research findings into an active treatment by targeting biological alterations consistently linked to PTSD diagnosis and symptom severity. If short-term mifepristone treatment is found to be effective and its effects persist beyond the period of medication intake, it may present a very different approach to pharmacological treatment than chronic psychotropic medication usage. This approach may be preferable to the many veterans with PTSD who do not want to be on psychopharmacological treatments continuously or long-term. Additionally if mifepristone can reverse the HPA axis dysregulation associated with PTSD and its associated behavioral, cognitive and immune alterations, strategies could be developed to favorably alter the long term course of this illness.

2.c. Relevance to the VA mission

PTSD is a highly prevalent disorder within the VA population. It is imperative that more effective treatments that lead to recovery from PTSD and its associated cognitive, social, and occupational impairments are developed. Given the high burden of illness associated with PTSD there is a compelling need to develop novel treatment approaches to reduce the enormous personal and societal costs of this disorder.

Fig. 2 Effect of mifepristone on GR sensitivity in subjects with and without PTSD

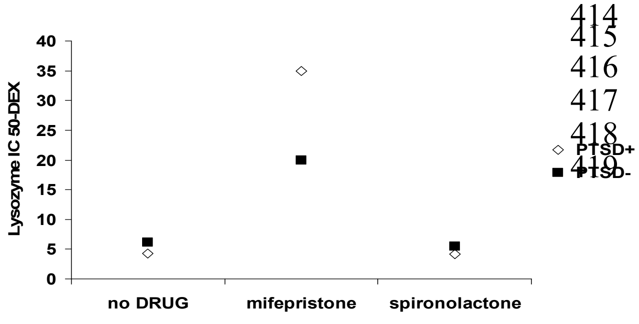

3. Work Accomplished

The central hypothesis of this application is that increased glucocorticoid receptor sensitivity is an important aspect of HPA axis dysregulation in PTSD. In

anticipating this trial, we first obtained information about the effects of mifepristone on peripheral GR sensitivity from participants with (n=8) and without (n=9) PTSD. Mononuclear cells were isolated from blood and divided into two portions. In one portion, cells were incubated with a series of concentrations of DEX. The other portion was incubated with the same concentrations of DEX and 10μM of RU486 (mifepristone). At baseline, the concentration of DEX at which lysozyme activity was inhibited by 50% (i.e., the lysozyme $IC_{50\text{-DEX}}$) was significantly lower in trauma exposed participants with PTSD compared to those without PTSD, indicative of increased peripheral GR sensitivity (4.5 ±1.5 nM vs. 6.1 ±0.9 nM; $F_{1,15}$=6.9, p=0.019). Co-incubation of cells with mifepristone and DEX robustly antagonized the glucocorticoid response as shown in Figure 2. Mifepristone significantly shifted the IC_{50} in response to DEX antagonized glucocorticoid responsivity on a measure that is altered at baseline in PTSD, providing evidence that this medication targets a peripheral biological characteristic associated with PTSD.

In order to gain familiarity with mifepristone, we obtained pilot funding from the VISN 3 MIRECC to study a small number of veterans with PTSD using similar inclusion/exclusion criteria to those that are outlined in this protocol. Eight combat veterans with military-related PTSD were treated in a randomized, double-blind trial of mifepristone (600 mg/day) or placebo for seven days and followed longitudinally for one month. The data for each clinical and neuroendocrine outcome measure are shown in Table 1. Based on the clinical response definition of a 12-point or greater decrease in total CAPS score from initial evaluation, all four veterans in the mifepristone group and one of four veterans in the placebo group achieved clinical response at one month follow-up. Similar improvements were observed using the clinical response definition of a 30% decrease in total CAPS-2 score. Three of four veterans in the mifepristone group achieved clinical response at treatment endpoint based on this definition, compared to one of four in the placebo group; these benefits were observed at one month follow-up, as two of four veterans in the mifepristone group maintained clinical response. Furthermore, between groups analysis revealed that the mifepristone group had significantly greater improvements in total CAPS score, CAPS avoidance symptom score, and PCL score at one month follow-up when compared to the placebo group. With respect to neuroendocrine outcomes, mifepristone induced significant acute changes in plasma cortisol (p=0.007), ACTH (p=0.009), and GR binding (p=0.041) from baseline to treatment endpoint; these findings are consistent with successful glucocorticoid receptor blockade. The acute decrease in GR number also points to the possibility increased cortisol binding and signaling. Moreover, this pilot clinical trial had a high retention rate and no adverse events or complaints about study procedures were reported, suggesting that mifepristone treatment is safe, tolerable, and acceptable to veterans with PTSD. Thus, these striking pilot data suggest that treatment with mifepristone is a feasible strategy that may be effective in improving clinical outcomes in veterans with PTSD.

Table 1: Clinical and Neuroendocrine Outcomes

		Pre-Treatment	Post-Treatment		Between Groups Significance	
		Initial	Endpoint	4-week	Initial to Endpoint	Initial to 4-week
		Mean (SD)	Mean (SD)	Mean (SD)	p-value	p-value
CAPS score	Drug (n=4)	68.75 (10.24)	43.50 (19.49)	44.00 (24.12)	0.425	**0.047**[a]
	PBO (n=4)	68.25 (8.77)	56.50 (31.89)	65.25 (18.86)		
Intrusive Sx	Drug (n=4)	13.75 (8.06)	8.00 (7.70)	7.50 (9.00)	0.463	0.126
	PBO (n=4)	14.50 (4.65)	12.00 (10.30)	15.00 (9.42)		
Avoidance Sx	Drug (n=4)	29.25 (7.14)	18.75 (11.44)	17.00 (10.74)	0.239	**0.027**[b]
	PBO (n=4)	28.50 (1.29)	25.50 (7.05)	26.00 (5.72)		
Hyperarousal Sx	Drug (n=4)	25.75 (0.50)	16.75 (5.56)	19.50 (9.68)	0.786	0.359
	PBO (n=4)	25.25 (6.40)	18.25 (10.53)	24.25 (5.97)		
BDI	Drug (n=4)	22.25 (5.85)	13.25 (4.72)	11.50 (9.15)	0.124	0.769
	PBO (n=4)	24.25 (12.12)	24.75 (10.05)	23.75 (9.54)		
PCL	Drug (n=4)	60 (10.83)	46.75 (11.79)	49.25 (15.59)	0.691	0.200
	PBO (n=4)	63.25 (11.41)	54.25 (24.17)	56.25 (16.68)		
Cortisol (μg/dL)	Drug (n=4)	13.15 (3.52)	31.28 (5.49)	11.53 (4.27)	**0.001**[c]	0.786
	PBO (n=4)	12.43 (6.16)	12.90 (4.87)	11.83 (2.91)		

ACTH (pg/ml)	Drug (n=4)	49.33 (29.69)	153.45 (40.25)	53.63 (8.08)	**0.009**[d]	0.785
	PBO (n=4)	30.88 (14.83)	38.40 (16.18)	41.63 (19.33)		
Lysosyme IC50 (nM)	Drug (n=2)	8.00 (2.55)	5.57 (.76)	6.73 (3.66)	0.162	0.411
	PBO (n=4)	3.27 (2.89)	3.65 (2.06)	5.03 (1.68)		
GR/cell	Drug (n=3)	1557.67 (539.75)	188.67 (116.89)	1593.00 (770.02)	**0.003**	0.322
	PBO (n=4)	1019.50 (553.91)	1243.00 (543.84)	1824.75 (683.80)		

[a]Median change in score for mifepristone group (20.0); median change in PBO group (10.5); non-parametric p-value (0.021)
[b] Median change in score for mifepristone group (11.0); median change in PBO group (4.0); non-parametric p-value (0.028)
[c] Median change in cortisol for mifepristone group (17.2); median change for PBO group (0.4); non-parametric p-value (0.021)
[d] Median change in ACTH for mifepristone group (90.6); median change for PBO group (4.2); non-parametric p-value (0.021)

4. Work Proposed

4.a. Overview

90 eligible male veterans with chronic PTSD will be randomly assigned to treatment with 600 mg/day mifepristone or placebo for one week and assessed for clinical outcomes at one and three months follow-up. Eligibility will be based on the inclusion and exclusion criteria which are enumerated below and in the Human Participants section. The inclusion and exclusion criteria were selected in order to include as representative a sample as possible while also addressing safety concerns. Veterans who are actively suicidal as assessed by the Columbia-Suicide Severity Rating Scale (C-SSRS) or who have attempted suicide within the past two years will be excluded. (If suicidality is identified, the necessary steps will be taken to ensure the appropriate clinical care is provided, and the local suicide prevention policies implemented.) Veterans with adrenal insufficiency will be excluded, as will veterans with other major medical or neurological illnesses, as they may be at increased risk of developing adverse events. Veterans with renal disease/impairment, hepatic disease/impairment, cardiac illness (e.g. coronary vascular disease, congestive heart failure), or hypokalemia at screening will also be excluded. Since mifepristone use can prolong the QTc interval in a dose-related manner, veterans with a prolonged QTc interval, defined as >450 msec, on the ECG at screening will be excluded. To evaluate QTc prolongation post-mifepristone treatment, an ECG will also be performed at both the 3 day follow up visit (visit 1.5) and 1 week follow-up visit (week 1, visit 2).

Participants on potent CYP3A4 inhibitors (fluconazole, ketoconazole, itraconazole, erythromycin, rifampin) and some anticonvulsants (e.g., phenytoin, phenobarbital, and carbamazepine) will be excluded since these medications impact the metabolism of mifepristone (see Risk/Benefit Assessment for details). Due to an increased risk of adverse drug reactions, veterans taking simvastatin, lovastatin, fentanyl, pimozide, bupropion, nefazodone, dihydroergotamine, ergotamine, quinidine, sirolimus, carvedilol, propanolol, diltiazem, verapamil, alprazolam, or tacrolimus will also be excluded. Since the impact of mifepristone on the male reproductive system has not been extensively studied, only veterans willing to use effective means of birth control for up to 90 days after mifepristone ingestion will be eligible; this will cover the critical period of fetal development.

For veterans prescribed psychotropic medications (i.e., antidepressants, antipsychotics or anxiolytics/sedative-hypnotics), the veteran is required to be on a stable dose for at least five weeks prior to screening. For veterans not taking psychotropic medication, a minimum of five half-lives must elapse prior to screening since the veteran last took any given psychotropic medication. Should it be necessary to adjust a patient's treatment regimen during the 3 month treatment period, standard pharmacological care for PTSD will be instituted. Major depression and other anxiety disorders are not exclusionary since they frequently co-occur with PTSD and it remains unclear whether their presence represents true comorbidity, symptom overlap, or severe illness. Since mild head trauma is common in the military population in general, excluding such participants would diminish the generalizability of the sample; participants with severe traumatic brain injury (TBI), defined as an extended period of unconsciousness or amnesia following injury, will be excluded. The Ohio State University (OSU) TBI Identification Method will be used to assess lifetime history of TBI. This method first establishes all significant injuries in one's life and then determines if a TBI may have occurred based on whether the participant experienced a loss of consciousness (LOC) and, if so, for how long. A person is said to have a mild TBI if LOC does not exceed 30 minutes for any injury, a moderate TBI if LOC is between

30 minutes and 24 hours, and a severe TBI if LOC exceeds 24 hours. The OSU TBI assessment will be administered by a trained rater during the psychiatric evaluation conducted at screening.

Additionally, veterans diagnosed with alcohol/substance abuse and dependence will be excluded only if they are recently engaged in a maladaptive pattern of use or abuse. More specifically, persons who meet diagnostic criteria for alcohol/substance dependence will be excluded if they have manifested dependence within the previous three months (i.e., have met three or more of the seven criteria for a maladaptive pattern of use in the last three months). Persons with alcohol/substance abuse (who, by definition, do not meet criteria for alcohol dependence) will be excluded if they have shown a maladaptive pattern of alcohol use during the past one month (i.e., have met one or more of the four criteria for a maladaptive pattern of abuse).

Veterans who are currently receiving psychotherapies - individually or in a group setting - that are considered to have significant benefit for PTSD, according to the VA and DoD's Treatment Guidelines, will also be excluded. These therapies are cognitive therapy for PTSD (e.g. cognitive processing therapy (CPT)), exposure therapy (e.g., prolonged exposure therapy), stress inoculation training, and eye movement desensitization and reprocessing (EMDR). Other forms of therapy and case management which do not specifically target PTSD symptoms and/or have not been shown to provide significant benefit in PTSD will be allowed at entry and throughout the study (e.g. supportive therapy, psychodynamic therapy, anger management, cognitive behavioral therapy for symptoms or problems other than PTSD).

We recognize that PTSD in women is increasingly common and effective treatments are needed for this group as well. However, women will be excluded from this initial trial for safety reasons, since mifepristone is an abortifacient. Should mifepristone prove to be effective in male veterans with PTSD, this would provide a rationale for future studies in women, employing additional safeguards.

4.a.1. <u>Inclusion Criteria</u>

1. Participant is a male veteran.
2. Veteran meets DSM-IV diagnostic criteria for chronic PTSD.
3. Veteran has a CAPS total score (past month symptom status) greater than or equal to 50 at screening.
4. For veterans taking psychotropic medications (i.e., antidepressants, antipsychotics or anxiolytics/sedative-hypnotics), the veteran will be on a stable dose for at least five weeks prior to screening.
5. For veterans not taking psychotropic medication, a minimum of five half-lives must elapse prior to screening since the veteran last took any given psychotropic medication.

<u>4.a.2.Exclusion Criteria</u>

1. Veteran recently continued to engage in a maladaptive pattern of alcohol/substance use and/or abuse (as defined in protocol).
2. Veteran has used potent CYP3A4 inhibitors (fluconazole, ketoconazole, itraconazole, erythromycin, rifampin) and inducers within five half-lives prior to randomization.
3. Veteran is taking simvastatin, lovastatin, fentanyl, pimozide, bupropion, nefazodone, dihydroergotamine, ergotamine, quinidine, sirolimus, tacrolimus, or clarithromycin, cyclosporine, St. John’s Wort, diltiazem, verapamil, propranolol, alprazolam, carvedilol or some anticonvulsants (phenytoin, phenobarbital, or carbamazepine) within five half-lives prior to randomization.
4. Veteran is taking oral corticosteroids within five half-lives prior to randomization.
5. Veteran should be free of a major medical illness and medical condition that contraindicate the administration of mifepristone. These include but are not limited to:
    a. Veteran has a history of adrenal insufficiency or a low plasma cortisol level at screening (a.m. level less than 5 mcg/dl or a p.m. level of less than 3 mcg/dl.)
    b. Veteran has a history of severe traumatic brain injury, a history of a stroke, or another neurological illness or injury likely to impact cognitive functioning.
    c. Veteran has diabetes mellitus, an endocrinopathy, or another major medical illness.

d. Veteran has a history of cardiovascular disease including a history of angina, myocardial infarction or other evidence of coronary artery disease, or congestive heart failure
e. Veteran has prolonged QTc interval >450 msec on ECG at screening.
f. Veteran has hypokalemia at screening (defined as potassium level < 3.5 mEq/L)
g. Veteran has a history of hepato-biliary disease or an AST, ALT greater than 2X the ULN.
h. Veteran has a history of renal disease or an estimated GFR of < 60 ml/min.

6. Veteran has a lifetime diagnosis of schizophrenia, schizoaffective disorder, or type I bipolar disorder.
7. Veteran has a history of attempted suicide within the previous two years or active suicidal ideation within the past month as assessed by the Columbia-Suicide Severity Rating Scare (C-SSRS).
8. Veteran is currently receiving specialized trauma-focused psychotherapy, such as prolonged exposure therapy and cognitive processing therapy.
9. Veteran is not willing to use effective means of birth control during the study.
10. Veteran has a history of allergic reaction to mifepristone.
11. Veteran is found to be unsuitable for study participation at the discretion of the site investigator for any reason.

4.b. Recruitment Process and Informed Consent

Patients with a presumptive diagnosis of PTSD will be recruited through direct contacts during visits to outpatient mental health and primary care clinics by informing mental health care providers and other clinical providers about the study so as to elicit direct referrals and, having obtained Institutional Review Board (IRB) approval for a partial HIPAA waiver, by direct contacts after chart reviews and physician consultations for patients with PTSD. Before contacting potentially eligible patients, the study team will review the clinical history in the electronic patient chart at the site to investigate inclusion and exclusion criteria. For those eligibility items not found in the chart or found but out-of-date, the study team will determine what information needs to be obtained during the pre-consent and post-consent screening visits. Once it is mutually agreed upon that the patient is eligible for trial participation, that the strategy within the protocol is clinically acceptable, and that the primary physician is agreeable to the patient being contacted for possible study participation, patients will be contacted. The method for this contact, (i.e. by phone or letter and with pre-established oral or written scripts) will require approval by the governing IRB. If patients from any of the above contact sources agree to be evaluated, they will have an initial visit with the study team. During this visit, the study team will ensure the patient fits the study's inclusion criteria and does not have a reason to be excluded. The study will be explained to them and the Informed Consent Form (ICF) will be reviewed in its entirety. At that time, or after further consideration of the study by the patient and his physician and family, the patient will be asked to sign the ICF and HIPAA authorization, copies of which will be given to them. The investigators consider this consent process to be an ongoing process maintained throughout the study to provide patients with a continued understanding of the protocol, their participation, and their rights as human research participants. Remuneration is provided for time and expenses ($25 for screening/medical evaluation; $50 per visit for a total of four visits (baseline, one week follow-up, one month follow-up, three month follow-up)), but is not so high as to coerce an individual to participate who would not otherwise participate (see Human Subjects section for more information on participant remuneration). After the signed ICF is obtained, additional screening procedures covered in the ICF will be conducted prior to randomization.

Participant recruitment will be the responsibility of the clinical research staff and local principal investigator (PI). The study staff will educate their clinical colleagues regarding this study opportunity. Veterans with a confirmed or presumptive diagnosis of PTSD will be identified in VA outpatient clinics (e.g. PTSD, mental health, or primary care clinics). Mental health professionals at the VA will be alerted about the study and the study staff will maintain frequent communication with them so that potentially eligible veterans will be referred to the study. Veterans expressing an interest in learning more will be referred to study personnel for preliminary screening and informed consent. Informed consent will be obtained by the study PI or by an IRB-approved delegate after a screening interview, but prior to the initiation of any study procedures. As the initial step in the consent process, the study will be explained to the veteran in detail and the consent

form will be read to the veteran in its entirety by the PI or an IRB-approved delegate. The participant will be fully informed about all aspects of this study and will be given ample time for clarification and to have his questions answered. The veteran will be able to bring the consent form home and can discuss the study with his physician, family members, or others prior to making a decision. A de-identified log will be kept of all veterans who are screened for the study, even if they are ineligible, in order to characterize the larger population from which the sample was drawn and the reasons veterans were found to be ineligible.

4.c. Screening and Baseline Assessment

Informed consent will be obtained prior to the initiation of study procedures utilized for the purpose of determining eligibility. In order to determine eligibility, a psychiatric and medical assessment will be performed to make a diagnostic determination regarding PTSD status and to evaluate the presence or absence of exclusionary medical and psychiatric conditions. The health assessment will consist of a medical history (including current symptoms, history of medical illness and hospitalizations, medication use), physical examination (with measurement of vital signs, weight, height), and laboratory testing (complete blood cell count, fasting glucose, electrolytes, magnesium, creatinine, thyroid stimulating hormone, liver function tests, lipid profile, urinalysis, cortisol and electrocardiogram (ECG)). Psychiatric interviews will be performed by a trained psychometrician using the CAPS and the Structured Clinical Interview for DSM-IV (SCID) in order to accurately classify PTSD and associated psychiatric comorbidities and to identify exclusionary psychiatric illnesses. Participants will also be given the OSU TBI Assessment, Columbia Suicide Severity Rating Scale (C-SSRS) (Posner et al., 2008), Miller-Forensic Assessment of Symptoms Test (M-FAST) (Veazey et al., 2005), Combat Exposure Questionnaire (CEQ), Neurobehavioral Symptom Inventory (NSI), and Trauma Assessment which will further assess their eligibility for the study. Once a participant is determined to be eligible, he will be contacted by the clinical research assistant to schedule a baseline assessment. Baseline visits will begin at 8:00AM (± 1 hour).

Blood samples will be obtained for the assessment of basal pre-treatment neuroendocrine activity (i.e., plasma cortisol and ACTH levels). Veterans will be called the day before their scheduled visit and asked to fast from midnight until after the blood draw.10 ml of blood will be drawn by routine venipuncture for the determination of basal plasma cortisol and ACTH. Blood samples for hormonal analysis are collected into tubes containing ethylenediaminetetraacetic acid (EDTA), spun immediately in a cold centrifuge, and frozen for subsequent analysis. They will be shipped in batches to the Core Lab. Since observed changes in ACTH and cortisol could effectively break the blind if performed in the local hospital lab, samples will be assayed at the Clinical Neuroscience Lab at the James J. Peters VA Medical Center (Director: Dr. Yehuda).

Clinical assessments to be administered at the baseline visit include the CAPS, Beck Depression Inventory (BDI) (Beck et al., 1996), PTSD Checklist (PCL Military Version) (Weathers et al., 1996), National Institute on Alcohol Abuse and Alcoholism (NIAAA) Quantity and Frequency Questionnaire, State-Trait Anger Expression Inventory (STAXI) (Spielberger et al., 1988), and the Pittsburgh Sleep Quality Index (PSQI) (Buysse et al., 1989).

To address quantity and frequency of alcohol use within the last 30 days, participants will be asked to answer three NIAAA questions. This information will be obtained at baseline, 1 week follow-up, 1 month follow-up, and 3 month follow-up. Participants will be asked to report how many days per week or days per month they had at least one alcoholic beverage (defined as 1 can or bottle of beer, 1 glass of wine, 1 can or bottle of wine cooler, 1 cocktail, or 1 shot of liquor) in the last 30 days, how many drinks they consumed on average on the days in which they drank, and how many times during the last 30 days they consumed 5 or more drinks on one occasion. This measure will be used as a potential covariate to examine the effects of alcohol use on clinical outcomes.

Since grapefruit juice may increase the amount of mifepristone in blood and increase the chance of adverse events, participants will be instructed to not drink grapefruit juice while they are taking study medication.

Participants will be randomized at the end of the baseline visit, and will receive study medication.

4.d.Randomization

Prior to randomizing a patient, all screening and baseline evaluations must be completed and the patient determined to be eligible for inclusion in the study. Randomization should occur no later than two weeks from the completion of the screening visit.

4.d.1. *Randomization Methods*

The randomization scheme and associated codes will be developed by the Hines Cooperative Studies Program Coordinating Center (CSPCC). Permuted block randomization will be employed to assign patients to 600 mg mifepristone or placebo. Randomization will be stratified by study site.

4.d.2. *Randomization Procedures*

Patients determined to be eligible and willing to participate in the study will be randomly assigned to 600 mg mifepristone or placebo using a web-based randomization system with a telephone call randomization system as back-up. The clinical research assistant at the participating facility will be required to sign into this password-protected site. The clinical research assistant will enter the patient study number and will be asked to answer a few questions about eligibility in order to complete the randomization procedure. If met, the website will select the first unused entry from the pre-specified list of random treatment assignments for the particular site and then assign a randomization number, which will be used to order the double-blind treatment prescription from the study site pharmacy. The randomization lists are stored on a secure server at Hines CSP Central Study File labeled "restricted access". A copy of appropriately executed and completed consent and HIPAA authorization documents must be on file at the Hines CSPCC within 24 hours of randomization, preferably before the patient is randomized. If the internet server is down during normal business hours, the clinical research assistant can telephone the Hines CSPCC to randomize a patient.

If a participant hasn't received the study drug on the day the participant is randomized, site coordinators should make every effort to ensure the participant receives the drug within 3 days post randomization, follows the directions on how to take the drug correctly, and that no study prohibited concomitant medications are used during this period. Should the study drug assignment program be down on any business day, site coordinators will contact the Albuquerque Pharmacy Center to request an emergency manual assignment.

4.e.Treatment Algorithm

Medication treatment will be for one week duration, and randomized participants will be given a seven-day supply of medication. Three weeks after the pulse treatment, the participant will undergo clinical reassessment. If participants develop an AE or as SAE that is deemed unsafe by the PI, they will be taken off treatment immediately but will be followed per protocol. The research team does not anticipate the occurrence of study-related SAEs, as the dose levels used in this trial have been used before in other studies without SAEs. In our pilot study of mifepristone in PTSD, no veterans were taken off treatment.

Both the active study medication and its matching placebo will be donated by Corcept Therapeutics.

4.f.Follow-Up Assessments

The study physician will assume the psychiatric care of the participant for the duration of the study. A research flag in the electronic medical record will be established for each patient to alert other providers to the veteran's participation in the study and of the need to refer psychiatric matters to the study physician. The patients other providers will also be informed of important clinical events by personal communication and/or through their inclusion as additional co-signers on research-related notes made in the electronic medical record.

As outlined in Table 2, the veteran will be assessed in person 48 to 72 hours after taking the first dose of the study drug. An ECG, vital signs (pulse, blood pressure, temperature), and medical labs (complete blood cell count, fasting glucose, cortisol, and electrolytes) will be taken. Any symptoms and changes in concomitant medications will also be reviewed. The checkup is intended for safety reasons. Follow ups will occur immediately after the pulsed treatment (one week follow-up), at one month follow-up, and at three month follow-up. At each of these three visits, the participant will be assessed for safety (adverse event reporting, vital

signs and weight, C-SSRS interview), PTSD and psychiatric status (CAPS and self-report measures), and concomitant medication use. In addition, blood will be drawn for medical labs (complete blood cell count, fasting glucose, and electrolytes) and neuroendocrine assessment (plasma cortisol and ACTH) at the day 3, one week and one month follow-up visits; an ECG will also be performed at the one week follow-up visit. Refer to figure 3 for a timeline of study visits.

In order to quantify the amount of co-therapy the participant receives during the study, the psychosocial treatment schedule provided in the Longitudinal Interval Follow-up Evaluation (LIFE) study will be used. With this instrument we will collect data about the types of therapy the veteran has received. Specifically, we will collect information about the number of sessions per week that the patient has had of the following types of therapy: individual, group, family, medication session, or self-help. The information will be collected at one month intervals (1 month follow-up (week 4, visit 4); 2 month follow-up telephone check-in (week 8, visit 6); 3 month follow-up (week 12, visit 8).

Additionally, the clinical research assistant will be responsible for contacting the study participant in-between visits via telephone (i.e., week 2 (visit 3), week 6 (visit 5), week 8 (visit 6), and visit 10 (visit 7)) so that contact will be made with the participant at least every two weeks. Week 2 has a ±2 day window for visit scheduling, while weeks 6, 8 and 10 each have a ±5 day window for visit scheduling. Through this telephone check-in, site investigators will be able to monitor safety, obtain PTSD symptom ratings, and identify any signs of deterioration. To assess PTSD symptoms longitudinally, the PCL will be administered at every in-person study visit and will be administered by telephone at each biweekly check-in. The study participants will be given a laminated card with the PCL on it to take home and will indicate their responses to the clinical research assistant who will record them; it is estimated that this would take ten minutes or less. This telephone check-in should also help enhance retention. A script will be developed for the telephone check-ins so they are standardized across time and site.

Table 2: Schedule of In-Person Assessments

		Pre-treatment		Post-treatment			
		Screening	Baseline	3 day follow-up	1 week follow-up	1 month follow-up (study endpoint)	3 month follow-up
Week			Week 0	Day 3	Week 1	Week 4	Week 12
Visit		0	1	1.5	2	4	8
Informed Consent		X					
Diagnostics	Demographics	X					
	Psych Diagnosis (CAPS month, and lifetime, SCID, and M-FAST)	X					
Medical	Medical History	X					
Safety	Vital Signs and Weight	X	X	X	X	X	X
	Medical Laboratory* (CBC with differential, fasting glucose, electrolytes, creatinine, liver function tests, magnesium, TSH	X (additional Lab on lipid profile, urinalysis, cortisol)		X	X	X	
	ECG	X		X	X		
	Adverse Events			X	X	X	X
	Concomitant Meds		X	X	X	X	X
	C-SSRS	X			X	X	X
Clinical Outcomes	PTSD Symptoms (CAPS past week, past month)		X		X	X	X

	Self-Reports (including PCL)		X		X	X	X
	Alcohol Consumption		X		X	X	X
Neuroendocrine	Plasma cortisol, ACTH†		X	X (only plasma cortisol will be collected**)	X	X	
	Plasma mifepristone and metabolites‡				X		

*Glucose, electrolytes, creatinine and liver function tests may be ordered together by requesting the comprehensive metabolic panel.

**For cortisol collection at day 3, the samples will be centrifuged on site, aliquoted into labeled cryovials, and stored frozen in a deep freezer (-80 degrees Celsius) until shipped in batches to the Core Lab. In the case of a significant AE/ SAE a cortisol determination will be made at the local lab.

† The blood tubes for neuroendocrine testing (cortisol and ACTH) will be centrifuged on site, aliquoted into labeled cryovials, and stored frozen in a deep freezer (-80 degrees Celsius) until shipped to the Core Lab.

‡ Blood samples of mifepristone and its metabolites will be de-identified and labeled with participants' study ID numbers, draw date, and draw time. The blood tube will be centrifuged on site, aliquoted into labeled cryovials for 1 ml of plasma, and stored frozen in a deep freezer (-80 degrees Celsius) until shipped to MicroConstants

Figure 3: Timeline of visits.

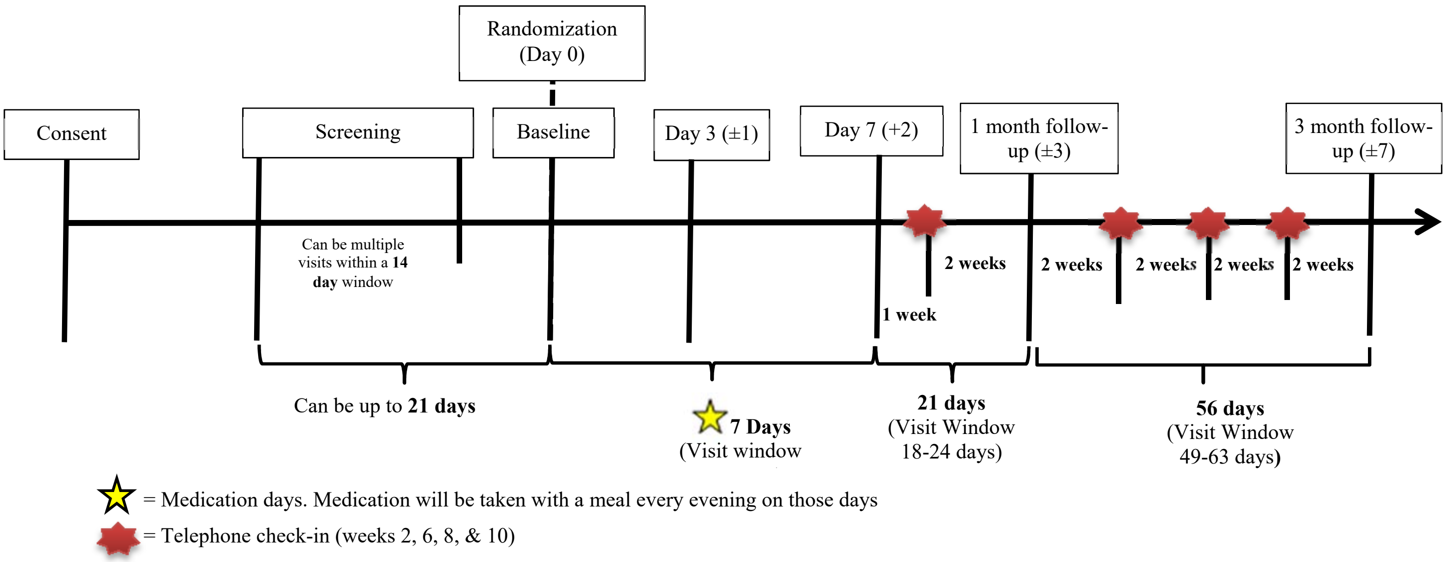

4.g. Treatment Adjustments

Since PTSD is a chronic disorder it is important that this study is of sufficient duration to assess the full impact of pulse treatment upon symptoms and the extent to which it is sustained; therefore, a three month study is proposed. However, since this is a placebo-controlled study, it may not be safe to delay pharmacological treatment or changes in treatment over three months in the face of increased symptoms or decreased functioning. Delaying treatment adjustments could also impact study integrity; if treatment delays led to a high number of "dropouts" it could compromise the interpretability of the study. Careful selection of participants without suicidality or a history of psychotic disorder and frequent monitoring during the trial should minimize the likelihood of treatment adjustments within the first month, and possibly throughout the

duration of the trial. Routine treatment adjustments during the 3 month treatment period will be discouraged, especially during the first month of enrollment in the study due to possible confounding impacts on the primary study endpoint. Nonetheless, should it be necessary to adjust a patient's treatment regimen during the 3 month treatment period, standard pharmacological care for PTSD will be instituted or adjusted at the discretion of the local PI. Treatment adjustments should preferentially be restricted to when there is a concern about clinical deterioration or significant decrease in functioning. Indications for treatment adjustment will include an increased potential for suicide or aggression, a significant increase in severity of PTSD or associated symptoms, or functional decline. If treatment adjustments are initiated, patients do not have to be withdrawn from the study. Careful application of the inclusion/exclusion together with the allowed use of psychopharmacology should minimize the use of treatment adjustments. The primary reasons that these medication adjustments are allowed are to prevent drop-outs of this study which can significantly harm the integrity of the study as well as to prevent bad clinical outcomes.

Instituting one of the structured psychotherapies for PTSD (e.g. prolonged exposure therapy, cognitive processing therapy) as a treatment adjustment would be problematic in so far as they can temporarily increase the severity of PTSD symptoms in some patients and can be quite effective in some patients. It would be difficult to account for these effects when interpreting the effects of mifepristone treatment. Thus, while specialized psychotherapeutic treatments are not strictly prohibited, the site PIs would need to discuss the implementation of such therapies with the Principal Proponent.

4.h. Post-Treatment and Post-Study Care

All participants who exit the study prematurely during the acute treatment period will be encouraged to come in for a final termination visit for the assessment of adverse events, vital signs, and medical laboratory testing. All unused medication will be collected and sent to the study site pharmacy. The veteran will also be asked to complete a final clinical assessment three weeks after their last dose of medication. If any medical complication has arisen, an appropriate medical referral will be made. At study endpoint, the patient will be informed that the active study period has ended and that they will resume routine clinical care. To facilitate the transition, the veteran's psychiatrist or other mental health care provider will be informed about study completion and of any clinically important developments that occurred during the study. The site PI will ensure that the veteran has an adequate supply of medication until that visit and will provide care as needed until routine care is re-established. The veteran will be informed at the outset of the study that continuation on mifepristone will not be available to them after the study period ends. To preserve the integrity of the study, we will wait until follow-up on all patients is complete and then break the randomization code on all patients. A communication will then be sent to the patient informing them if they were on active treatment or placebo.

4.i. Safety Monitoring

Steps taken to minimize risk include excluding veterans with major medical illnesses including adrenal insufficiency, diabetes mellitus, and a history of severe TBI. The initial health assessment will consist of a medical history and physical examination with measurement of vital signs, weight, height, and laboratory testing (complete blood cell count, fasting glucose, electrolytes, magnesium, morning cortisol, creatinine, thyroid stimulating hormone, liver function tests, lipid profile, urinalysis, and ECG). For clinical safety monitoring during the trial, complete blood cell count, fasting glucose, electrolytes, and vital signs will be measured during the 3 day follow-up and one week follow-up visits. At day 3, blood will also be collected for cortisol and stored frozen for subsequent analysis at the Core Lab, located at the James J. Peter's VA Medical Center. The day 3 cortisol sample will be analyzed in the same manner as the day 7 sample, in batches, after the participant completes all study procedures. In the event of a significant AE or SAE, a blood sample will be submitted to the local medical lab for cortisol determination, if deemed necessary by the Medical Monitoring Committee and confirmed by the Study Chair. At one week follow-up blood will also be drawn for plasma levels of mifepristone and its metabolites. Although previous studies have not found that one week of mifepristone is associated with adrenal insufficiency, because there is a theoretical risk of this, participants will be monitored for this possible adverse effect. Participants will be specifically assessed for the presence of signs or symptoms of adrenal insufficiency (malaise, fatigue, vomiting, nausea, orthostasis, hyperkalemia, hyponatremia, and eosinophilia) at baseline and at follow-up visits. All investigators will grade adverse events using version 4 of

Common Terminology Criteria for Adverse Events (CTCAE) and Common Toxicity Criteria (CTC). Common events known to be associated with mifepristone will be assessed using the reference toxicity table included in the study manual of operations to decrease the variability across investigative sites. Per the FDA's request, the investigators will explicitly inquire about the development of a rash since this is a known side effect of mifepristone. Medication will be discontinued if a rash develops or adrenal insufficiency is suspected; for the latter, a referral to internal medicine and/or endocrinology will be made. If the rash is severe or persistent, the veteran should be referred to a dermatologist.

With respect to suicidality, veterans with a history of attempted suicide within the previous two years or active suicidal ideation within the past month (participant endorses active suicidal ideation with any methods without intention to act (question 3)), as assessed by the C-SSRS, will be excluded. For all study participants, suicidality will be re-assessed at in-person post-treatment visits (one week follow-up, one month follow-up, and three month follow-up) using the C-SSRS. If the participant develops active suicidal ideation (as elicited from the C-SSRS or spontaneously reported) at any point in the study, he will be further assessed; the appropriate clinical intervention will be initiated and the local procedures for notifying the suicide prevention coordinator will be followed.

To assist in monitoring patient safety, the sites will be provided with an Investigational Brochure (IB) prepared by Corcept Therapeutics and a Drug Information Report (DIR) that is prepared and updated as needed by the pharmacy coordinating center study clinical pharmacist. The DIR provides a comprehensive review of the published literature regarding mifepristone safety data as used in the treatment of psychiatric disorders. The IB and DIR are submitted along with the protocol to the IRB, the DMC, and to the FDA as part of the IND as the safety information for the study. The statistical coordinating center then distributes the IB, DIR, protocol, and Drug Treatment and Handling Procedure manual to the study sites.

Concomitant medications will be recorded in terms of their daily dose, indication, and start and stop date. Study drug compliance will be monitored by pill counts of returned medication. In addition to the monitoring of individual patients at the local site, the study will be reviewed by the CSR&D Psychiatric, Behavioral Health & Neurologic Disorders Centralized Data Monitoring Committee.

Study participants will be monitored at each clinic visit (day 3, weeks 1, 4, and 12) and via telephone contact (weeks 2, 6, 8, and 10) by site investigators and coordinators for AEs and SAEs. Participants will be asked about any AEs or SAEs that have occurred since the previous visit or telephone contact, and vital signs will be obtained at all in-person visits. This frequent contact with participants, both in-person and via telephone, will enable site investigators to monitor safety, obtain PTSD symptom ratings, and identify any signs of deterioration. Information on all AEs and SAEs, including those related to the study intervention and those not related to the intervention, will be collected and recorded on the appropriate study event forms. All relevant information (e.g. onset of event, severity, outcome, extent to which it is or is not attributable to the drug or other study procedures) will be captured. Active monitoring of AEs and SAEs will begin as soon as a study participant signs the informed consent document and will continue through end-of-study for each participant. Instructions for contacting the VA National Suicide Hotline will be incorporated into the consent form and instructions for patients as part of safety monitoring. These safety data will be analyzed as described in the Analysis Plan (4.q.) and used to inform additional studies with mifepristone in the treatment of PTSD.

All SAEs require expedited notification, which is defined as the completion and submission of the SAE form in the Cooperative Studies Program electronic data capture (eDC) system within 72 clock-hours of a site investigator's initial awareness of an SAE. The VA Cooperative Studies Program Clinical Research Pharmacy Coordinating Center (PCC) will be responsible for evaluating all AEs for patient safety concerns. SAEs that are both related to the investigative treatment and unexpected will be reported to the FDA, CSR&D Director, and site investigators after review by the Study Chair, the PCC Director, and the CSPCC Director. The Hines CSPCC will generate tabulations of all AEs and SAEs for the Data Monitoring Committee (DMC) annually or on a more frequent schedule if requested by the DMC. The PCC will provide to the Hines CSPCC safety data coded per the Medical Dictionary for Regulatory Activities (MedDRA) for the DMC reports.

Additionally, criteria for stopping enrollment for a safety pause are to be implemented. If 30% of every projected 10 participants in either dose arm report an adverse event (possibly or definitely attributed to the study drug initiated by the site investigator) at a severity level of 3 or above, or if 20% of the projected 10 participants at a severity level of grade 4 or above, or if any occurrence of a grade 5 adverse event occurs,

participant accrual to that specific dose arm is temporarily paused until review by the Medical Monitor Committee (MMC). Events are graded as follow: 1 = mild side effect, 2 = moderate side effect, 3 = severe side effect, 4 = life threatening or disabling side effect and 5 = death. The Medical Monitor Committee (MMC) will be composed of a Hines CSPCC physician (Yvonne Lucero, MD), a QA/Safety Monitor (Thomas P. Koppes, RN), Clinical Research Pharmacist from CSPCRPCC (Marcel D. Bizien, PharmD.), Psychiatrist Consultant (Timothy Kimbrell MD), and the Study Chair (Julia A. Golier, MD) to determine the relatedness to study drug. If the MMC agrees that the adverse events are related (possibly attributed or definitely attributed) to the study drug, further review by the DMC will be required. If the adverse event is not attributable to study drug, recruitment will continue. Hines would keep track of the rates as cases are reported in as well as review the cumulative data after every 10 cases on the study drug. If the DMC is asked to review the data, their recommendation of whether the study can proceed or not will be forwarded to the Director, CSR&D, for approval.

4.j.Blinding

4.j.1. *Double-blinding and breaking the blind*

The PCC will prepare a code-envelope for each randomization number, and the envelopes will be distributed to the study sites at the start of the study. The site will be responsible for providing a safe location for the envelopes that is accessible 24-hours per day (generally in the pharmacy) in the event breaking of the blind is authorized. Breaking the blind may compromise the integrity of the study; therefore the blind should only be broken when knowledge of the study treatment is needed to determine appropriate care of a medical complication thought to be related to the study treatment.

4.j.2. *Provision for breaking the blind*

In most cases, if there is a concern about patient safety and the study treatment, the patient should stop taking the study treatment without breaking the blind. When required by medical necessity two methods to unblind participants will be available: 1) 24-hour phone support by a PCC Clinical Pharmacist; and 2) utilization of the code-envelopes provided to each study site as described above. Specific instructions for unblinding will be provided in the Drug Treatment and Handling Section of the Study Operations Manual.

4.k. Outcome Assessment

The primary clinical outcome measure will be clinical response status at study endpoint (week 4), defined as a 30% or greater reduction in CAPS total score from baseline to week 4. Response status at week 12 will also be assessed as a secondary outcome measure. Total symptom severity during the prior week will be measured using the CAPS (Blake et al., 1995), a well-validated scale widely used in outcome studies of PTSD. PTSD symptom severity is calculated by summing the frequency and intensity rating of each of the 17 PTSD symptom items.

The secondary outcome measures will be the change in CAPS score from baseline to 1 week, 4 weeks and 12 weeks, the presence of clinical response as defined above at 12 weeks, and the percentage of study drug related AEs or SAEs.

The descriptive clinical outcome measures will be change from baseline to week 4 and week 12 for the following: the three PTSD symptom sub-scale scores from the CAPS, self-reported depression severity as measured by the BDI (Beck et al., 1996), PTSD symptom severity as measured by the PCL (Weathers et al., 1996), sleep quality as measured by the PSQI (Buysse et al., 1989), self-reported experience, expression, and control of anger as measured by the STAXI (Spielberger et al., 1988), and functional impairment as measured by item 24 of the CAPS.

Neuroendocrine outcomes include change from baseline to post-pulse treatment in plasma cortisol and ACTH levels. These will allow for assessment of the acute neuroendocrine response to 600 mg of mifepristone and its relationship to treatment outcome. Additionally, plasma levels of mifepristone and its metabolites will be assessed immediately after one week of treatment.

4.l. Study Conduct and Quality Control

4.l.1. *Study Conduct Monitoring and Oversight*

Figure 4. CCTA #0004 Organizational Structure

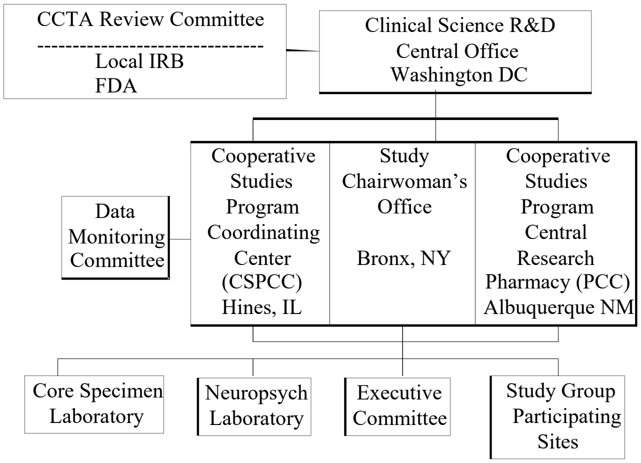

The groups charged with centrally monitoring the various aspects of the study will be the Executive Committee (EC), the CSR&D Central Data Monitoring Committee (cDMC), and the VA Institutional Review Board (VA-IRB). The EC is the management and decision-making body for all operational aspects of the study. It will meet in person at study start-up and annually thereafter, and will hold quarterly conference calls. One of its major responsibilities is to monitor the performance of the participating medical centers. The EC considers the need for protocol modifications, and also reviews and approves all manuscripts and abstracts emanating from the study. Typically, the EC is composed of the original study Planning Committee and is chaired by the Study Chair.

Prior to study initiation, the protocol and recruitment/consent materials will need to be reviewed for approval by the local site VA-IRBs. These bodies will provide a review for multi-center trials within the CSR&D, HSR&D, and RR&D branches of ORD. The local site VA-IRBs will also receive and review amendments, SAEs, and protocol deviations, and will perform annual continuing review certification. Finally, the study protocol and progress must be reviewed by the individual study site R&D Committees before site initiation, and annually thereafter.

Training will be provided before and at the kick off meeting for the CAPS and SCID, and the scoring conventions for this study will be reviewed in detail. Following the training session, all evaluators will send an audio-tape of their first two CAPS interviews for review by Dr. Janine Flory, the lead psychologist for this study, or a designated alternate. Feedback will be provided to the evaluators regarding their adherence to the structured format of the interviews and the use of follow up or clarification questions. The nature of this feedback will be qualitative.

Audio recordings of at least three CAPS interviews of male patients with PTSD will be sent to the sites for assessment of inter-rater agreement. The site interviewer's ratings and the study chair psychologist's ratings for total symptom severity (current) will be compared and an intra-class coefficient (ICC) will be determined. An ICC greater than or equal to 70% will be required. After reliability has been established, a random sample of 10% of the CAPS performed at each site will be selected and scored by each of the site interviewers for ongoing assessment of fidelity and reliability. Thus, each site will provide approximately 1 recording annually for ongoing inter-rater reliability.

An Olympus WS-802 personal digital recorder will be utilized by each assessor to record CAPS interviews. The WS-802 contains an external memory slot which enables users to copy audio files onto a microSD card. When not in use, all WS-802 digital recorders will be stored securely behind a locked door. Audio recordings that are selected for review will be transferred from the recorder to a microSD card (directly) and mailed to the Bronx VA via a traceable mail system (i.e., UPS) or courier service. These recordings will not contain PHI and files stored on the card will not be indexed by any identifiable means. Once received, the files will be transferred onto an IRM-approved, non-VA computer, and deleted from the card. They will then be uploaded to the VA-approved secure SharePoint server which will only be accessible by few designated study assessor personnel via a privacy setting. The SharePoint site can only be accessed using VA-secured computers. Once the audio has been listened to and scored, it will be deleted from the computer. The microSD card that contained the audio file will be formatted and sent back to the site in which it originated from.

Participants who agree to be recorded will sign VA consent form 10-3203. Those who decline to be audiotaped will not be excluded from the study. A telephone conference call will be held every one to two months, depending on volume, with the site interviewers and Dr. Flory to discuss CAPS scoring, consensus diagnoses, and to review difficult clinical cases.

The study DMC will be the CSR&D Psychiatric, Behavioral Health & Neurologic Disorders Central Data Monitoring Committee. The cDMC will initially meet before or at study start-up, determining at the first meeting the frequency of progress reviews required for the study, but will meet no less than annually. The cDMC will review the progress of the study including patient intake, completeness of follow-up, data quality, protocol deviations, and safety, and will also review any protocol modifications recommended by the EC. The cDMC will intermittently review data provided by study statisticians. The cDMC will make recommendations to the Director, CSR&D, through the Director, Hines CSPCC, as to whether the study should continue, be placed on probation, or be terminated.

The Study Group, which consists of all participating investigators and site clinical research assistants and assistants, will meet annually to discuss the progress of the study and any problems encountered during the conduct of the trial. Study personnel will adhere to ORD policy on human subjects' protections by fully completing approved Good Clinical Practices (GCP) training per VHA Handbook 1200.05. Completed training certificates will be provided by personnel to Hines Coordinating Center to maintain on file throughout the study. SMART (Site Monitoring, Auditing, and Resource Team), a division of the CSP Clinical Research Pharmacy Coordinating Center (CSPCRPCC), may conduct a full audit of participating sites if requested by the sponsor (CSRD). The study will be monitored utilizing centralized data and statistical monitoring methods and remote-based source verification; which will be based on risk assessment and management strategies.

4.l.2. *Training and Quality Control Measures to Assure Accuracy, Precision and Validity of the Data*

Toward the end of the start-up period, training of study personnel will take place during a kick-off meeting. All participating investigators, site clinical research assistants, and CSP coordinating center personnel will attend. Prior to the meeting, case report forms will be finalized and a detailed manual of study operations will be written and circulated. This manual will serve as the training manual for the meeting and as a reference document following the meeting. The study Chair's office (consisting of Dr. Golier, a research psychologist, and clinical research assistant) and the data and pharmacy coordinating centers will provide the training. The general training will include the study treatment, patient screening and consent, baseline evaluation, follow-up procedures, proper entry and maintenance of data, and AE/SAE safety reporting. In a special training session (see below), study clinical research assistants from all participating sites will be trained in the administration of a variety of state-of-the-art assessment instruments by the research psychologist to ensure accuracy and precision of administration of the instruments and to maximize inter-rater reliability. Training on the CAPS and SCID will be performed at the initial kick-off. A formal training on the CAPS and SCID will be provided at the kick-off meeting by the research psychologist who is an expert rater and who will establish inter-rater reliability. Inter-rater reliability coefficients of greater than 0.70 will be established for the total CAPS score. Quarterly supervision on the CAPS administration will be provided to ensure continued integrity of these assessments and inter-rater reliability will be re-assessed annually. Replacement staff will be trained as needed.

4.l.3. *Site Performance*

Hines CSPCC will evaluate recruitment and retention performance monthly. Other performance problems such as protocol deviations, poor data quality, missing or overdue data, and reasons for withdrawal from the study will also be tracked. A monthly study conference call for study personnel including Hines CSPCC, PCC, Chair's office, and all sites will be held to review study performance. Study sites will be put on probation for poor performance, including under-recruitment. Typically the probationary period is three months, at which time the site may be taken off probation, have probation continued, or have funding stopped, depending on its performance during the probationary period. The Hines CSPCC Director is authorized to make those decisions.

4.m. <u>Data Collection and Management</u>

4.m.1. *Data Collection*

The Hines CSPCC will manage the trial data using a web-based eDC system. Paper source documents will be provided to clinical research assistants as a primary means of collecting data. After a patient consents to participate in the study, the site clinical research assistant will create a patient casebook, which will contain the consent forms, all relevant source documents, and any other information pertinent to the study. The case

report forms will be completed at each visit through the Cooperative Studies Program's password-protected eDC system, accessed via the VA intranet. The eDC system will allow clinical research assistants to enter the source document data directly into a web-based study database and thus manage their patient's study activity, handle data clarifications, and correct patient data online. Accordingly, the electronic system will be used to create, modify, maintain, and retrieve clinical data for CCTA #0004 during each step of data collection including: 1. Initial screening, 2. Baseline visits and Randomization, 3. In-person study visits, 4. Follow-up telephone contacts, 5. End-point data collection, 6. Adjudication of endpoints and 7. Unscheduled visits for AEs/SAEs, Concomitant medications, Protocol Deviations, and Termination.

After the study is approved, the Study Chairman, Study Biostatistician, Study Project Manager and Study Pharmacist will prepare an Operations Manual for site staff to guide them through the operation and management of the study and data collection tools. A training session will occur at the study kick-off meeting for all investigators and clinical research assistants to assure uniformity in patient management, data collection, study procedures, and Good Clinical Practices. At this training, clinical research assistants will be provided with reference materials on the software tools and tasks. Once formal training is completed, user accounts – utilizing a URL specific to the study to access and use the system and enter patient data – will be activated. Accounts will be password protected and unique to the users' functional study group (i.e., those for a clinical research assistant would differ from those of the coordinating center or site monitors). Formal training on the use of the eDC system for clinical study management will also be provided. Systems training will also be held at annual meetings and on an as-needed basis for new study personnel. The study personnel, including the biostatistician, statistical programmer, database programmer, and data coordinator at Hines CSPCC, will have access to read the data.

CSP has developed an eDC tool that is fully compliant with US Federal regulations regarding electronic web-based data capture systems established by the Food and Drug Administration under 21 CFR 11. This system is designed to make the process of patient data management easier, timelier, and more efficient. It is accessed via the VA intranet. The paper source documents from which data will be entered into the electronic data capture tool's case report forms provide the official clinical record for data collection. All paper-based study records will be kept under lock and key. The eDC system will be validated by the Hines CSPCC Validation Team to ensure the integrity of the data capture software. Validation documentation and all system dependability documentation (i.e., software and hardware versions, etc.) will be maintained as study documentation, but have not been detailed in this protocol.

For this trial, eDC designers will create a study-specific database that includes case report forms, interview schedules, and data queries using customized C# code imbedded directly into Microsoft InfoPath (2007 or later) forms housed on a VA approved SharePoint Server Farm (2007 MOSS or later). Data queries will be managed in two ways. Certain queries will be programmed into the forms that are designed to activate upon data entry. Additional queries will be programmed using other data analysis tools such as SAS and will be uploaded into the system for clinical research assistants to address. Furthermore, the system will allow manual queries to be entered into the forms by the coordinating center as needed. Updates to the electronic forms and database can be generated during the study without impacting collected data. Study reports can be generated from exported data in order to track the study progress and to monitor adverse events, in particular SAEs. Study reports will be circulated to appropriate members, including the Site Investigators, the Study Chairman, and the Data Monitoring Committee (DMC).

4.m.2. *Data Quality Assurance.*

Electronic data capture methods will be used for the proposed study. The data will be entered by site clinical research assistants remotely through electronic case report forms. Extensive data checks, including missing values, out-of range entries, and consistency between variables, both within and across forms, will be built into the computerized data capture system developed for this project. There will be two levels of checking. The first level will be done at the time the data is entered into the case report form. These checks will automatically appear on the screen at the time of entry. After submission of the form into the database, second-level checks against other data already captured for that patient will be done. These checks will generate data discrepancies, which will be stored in the eDC system for the site clinical research assistant to access and rectify. Any revisions to the data made by the study site personnel will be recorded in a system audit trail. The

Hines CSPCC will monitor completeness and timeliness of the data discrepancy resolution made by site clinical research assistants.

4.m.3. *Data Confidentiality and Data Security.*

Handling and storage of study data will adhere to current VA policies. The database will not contain information that can directly identify the study participant (such as name, address, etc.); however, it will not be a completely de-identified database since age and study visit dates will be recorded. The Hines CSPCC requires that a copy of the signed consent form, HIPAA waiver, and a patient contact sheet be on file at CSPCC. The consent form is required by CSP policy in order for the coordinating center to independently certify that all study patients have been adequately consented in order to be randomized into the study. Patient contact sheets are collected in the event study participants need to be contacted (such as for safety notices) after study sites have completed the study. The Hines CSP Coordinating Center is the final repository of all study data and the only component that remains open and funded after the study ends. In the event that a study participant should need to be contacted well after the end of the study, his name, address, and phone number will be vital to the CSP center. Each patient's SSN is also necessary in the event that the Coordinating Center should have to access the Beneficiary Identification Records Locator (BIRLS) files to locate a patient. The electronic information from the contact sheets is stored separately from the main study database in a password-protected file. Any paper versions of the contact sheets are stored in locked file cabinets separate from other study data and accessible only by authorized CSPCC study staff. Consent forms will be faxed to a secure fax machine at Hines CSPCC. Contact form information will be entered into the electronic system as password-protected files.

Two mirror-images of the study database will be housed on separate servers located at different secure VA facilities that support round-the-clock web services and monitoring within a secure VA environment in order to provide an optimal infrastructure for the protection of sensitive information. Data entered by the site clinical research assistant is sent to the production server which is then copied, almost continuously to a back-up server at a different location. The clinical database with all research data will be housed behind the VA firewall on VA owned and maintained servers. Accordingly, the information housed within the eDC system will be afforded the same level of security as all forms of VA protected and/or highly sensitive information. Additionally, the system will be monitored by the Hines CSPCC Quality Assurance and Information Technology teams to ensure that all applicable VA regulations and directives are strictly followed.

Backup copies of the database will be transferred behind the VA firewall to the Hines CSPCC on a frequent basis depending on the study need (at least once per day). These backup copies will be transferred and stored across secure connections according to VA regulations and Hines CSPCC operating procedures. Periodic off-site back-ups will be made as part of a comprehensive disaster recovery plan. The Director of Information Technology will ensure that backup media are stored in compliance with all federal and VA regulations on the storage of potentially sensitive information. The Director of Information Technology will also ensure that all backup media is encrypted in compliance with the current best practices established and approved by the Center Director(s). Encrypted backup media will be stored in a physically secure location with access restricted to essential personnel. Access to back-ups may be at the discretion of the Center Director(s) and/or the Director of Information Technology.

Access to the study data is heavily restricted to individuals with CSP approval. Individuals must be properly credentialed research staffs who are compliant with VA security trainings (i.e., Research Data Security, HIPAA and VA Privacy Training, Information Security Awareness, and Good Clinical Practices). In addition, research data will be stored on VA secure servers with restricted permissions for copying and exporting data. Only properly approved coordinating center personnel will have the ability to copy and export data. These individuals have received training on the local standard operating procedures (SOP) governing their permissions and will not access or export data without approval from the Hines CSPCC Center Director. Furthermore, the permissions of the electronic systems are structured such that individual sites can only see the data for their study participants. They cannot see or access the data for another clinical site or for another participant.

Access to protected health information (PHI) will be heavily restricted to individuals approved by CSP to have access to the data. Approximately 18 staff positions will have access to some form of PHI for the study.

At the Local Clinical Sites, the following staff positions will have access to PHI. Individuals in these positions will be able to access all forms of PHI: A. Site Investigator, B. Co-Site Investigator and C. Clinical Research Assistant.

At the Hines CSPCC, the following ten staff positions will have access to PHI. Individuals in these positions will be able to access all forms of PHI: A. Center Director, B. Study Director, C. Project Manager, D. Data Manager, E. Biostatistician, F. Junior Biostatistician, G. Quality Assurance Officer, H. GCP Nurse Specialist, I. SAS Programmer, and J. Research Assistant.

At the CSPCRPCC, the following four staff positions will have access to PHI. Individuals in these positions will be able to access de-identified forms of PHI: A. Clinical Monitors, B. Study Pharmacist, C. Adverse Event Specialist (Regulatory Affairs and Safety Officer) and D. Pharmacy Project Manager

Research data will only be stored on secure VA servers within the VA firewall. Data will not be stored on desktops or on University affiliate servers. Study data will be coded with a unique study identifier for each participant and stored in a de-identified manner. Identifiable information will be collected for patient tracking and safety purposes. All private information will be kept on an encrypted, password-protected server to which a small number of people will have access. Access to the cross-walk file linking the participant's identifiers and their study data will be restricted to the clinical site and to the approved personnel at the coordinating center. This file will be destroyed according to CSP policy.

While the study is on-going, the electronic data capture systems will utilize state-of-the-art technologies in order to protect the data during transmission. All of these technologies exceed the current VA standards for transport. In brief, electronic systems will employ secure socket layer technology and FIPS 140-2 compliant encryption algorithms to ensure that data is not vulnerable during transport. In addition, all data will be stored within the VA firewall and will be password protected at all times. Hard copy data will be sent via a traceable mail system (i.e., UPS), via a courier, or via secure fax. Faxes are electronically routed to document management systems housed on VA protected servers located at the Regional Data Center in Philadelphia, PA. Access to these secure fax servers is restricted to the coordinating center personnel with approved access to the system. All secure fax servers are compliant with VA directive 1605.1 and 6500. All data security incidents will be reported in accordance with VA policy within one hour of discovering the incident to: 1) the District (local) Information Security Officer (ISO); and 2) the VA IRB. Quality control checks and clinical monitoring will enable the coordinating center to survey the study database and the clinical sites to ensure that the data have not been improperly used or accessed. 21 CFR part 11 compliant audit trails and access logs will be checked routinely. In addition, the clinical monitors will provide continuing education on Good Clinical Practices and will check all clinical site operations for violations of data security policies and best practices.

In order to ensure on-going reliability and fidelity of CAPS assessment, the study-specific SharePoint site will be used to securely share 10% of randomly selected CAPS audio recordings. Recordings selected for review will be transferred from the audio recorder to a microSD card (directly) by study personnel at each site and mailed to the Bronx VA via a traceable mail system (i.e., UPS) or courier service. These recordings will not contain PHI and files stored on the card will not be indexed by any identifiable means. Once received, the files will be transferred onto an IRM-approved, non-VA computer, and deleted from the card. They will then be uploaded to the VA-approved secure SharePoint server which will only be accessible by few designated study assessor personnel via a privacy setting. The SharePoint site can only be accessed using VA-secured computers and is regulated by the SharePoint administrators at the Hines VA. Once the audio has been listened to and scored, it will be deleted from each user’s workstation. The microSD card that contained the audio file will be formatted and sent back to the site in which it originated from via a traceable mail system (i.e., UPS) or courier service.

The clinical data for CCTA #0004 are considered property of the Cooperative Studies Program and shall not be sent off-site (i.e., outside of the Hines CSPCC) without the expressed, written permission from the Hines CSPCC Center Director and CSP Central Office. All data transfer and data security policies of the Cooperative Studies Program will be closely followed.

Retention and destruction of data will be conducted according to CSP operating procedures, as well as federal and local VA regulations. This will include paper and electronic data stored at the local sites, the Hines CSPCC, and at the VA facility housing our servers. Identifiable data will be kept according to CSP policy as outlined in the "CSP Guidelines for the Planning and Conduct of Cooperative Studies." Specifically, identifiers

will be kept on site at the Hines CSPCC for a minimum of five years or as dictated by the FDA or other regulatory agency with specific written procedures (i.e., two years after last approval of a marketing application, etc). At the end of the record retention period, the Hines CSPCC will conduct a review to determine if it is appropriate to archive the study data. If it is determined that the study data must be kept active, the Hines CSPCC will retain the database in its entirety until the primary and secondary analyses are completed. If the study is archived, the study data will be stored indefinitely. Study records maintained at the local sites cannot be destroyed without written permission from Hines CSPCC.

4.n. <u>Biostatistical Considerations</u>

The main aim of this study is to evaluate the potential efficacy of 600 mg mifepristone vs. placebo using a signal detection approach commonly used in Phase IIa drug-development trials. The approach was first specified by Simon et al. (1985) and falls within the class of ranking and selection procedures (Gibbons, Olkin and Sobel (1977)). Specifically, a response rate difference that is considered clinically relevant is selected (15% in this study). The sample size is based on providing a pre-specified degree of certainty (90% in this study) that if the response rate difference is observed, the treatment group with the higher response rate has been correctly identified. The analysis of the primary outcome then becomes a binary decision rule (i.e. has the pre-specified response rate difference been observed (yes or no)). The mifepristone 600 mg/day will be compared to placebo over a short duration, as is commonly done in Phase IIa trials. The advantage of using such design for this study is to detect the signal for comparing the 600 mg group vs. placebo while keeping the sample size small. The results generated from this study will provide the basis for a larger, more definitive, Phase III trial when type I error and power are well controlled.

4.n.1. *Expected treatment effect*

The objective of the current study is to assess whether mifepristone 600 mg is potentially effective for PTSD population. Currently, no published studies have examined mifepristone for the treatment of PTSD; however, other major drug studies in PTSD have defined responder status as a 30% reduction in CAPS score. Brady et al. performed a large multisite 12 week, double-blind, placebo-controlled trial to test the efficacy of sertraline; using conservative last observation carried forward, response rates were 53% for the drug group and 32% for placebo group (2000). Similarly, using the intent-to-treat principle, Davidson et al. reported response rates of 60% for the drug group and 38% for the placebo group (2001). The response rates of a double-blind, placebo-controlled pilot study of sertraline in military veterans with PTSD carried out by Zohar et al. were 41% for the drug group and 20% for the placebo group (2002). Marshall et al. evaluated the efficacy and safety of paroxetine for the treatment of patients with chronic PTSD; responder rates were 67% for the drug group and 37% for the placebo group (2001). A similar study carried out by Tucker et al. for paroxetine showed response rates of 58.8% and 38% for the drug and placebo group, respectively (2001). A large study of fluoxetine had response rates of 59.9% for the drug group and 43.8% for the placebo group (Martenyi et. al, 2002).

This study will use a 30% reduction in CAPS total score from baseline to four week follow-up to classify the participants as responders vs. non-responders. The proportion of responders to 600 mg mifepristone will be compared to the proportion of responders to placebo. The referenced studies report response rate differences ranging from 16% to 30% and placebo response rates ranging from 20% to 44%. In the present study, we will consider a 15% difference in the proportion of treatment vs. control group responders to be clinically relevant, as this is the minimum response rate difference in the referenced studies. Since the design of this study is more rigorous, a conservative estimate of the clinically relevant treatment effect is appropriate.

4.n.2. *Sample size determination*

As mentioned above, the sample size has been calculated to provide at least 90% probability that mifepristone 600 mg has been correctly identified as being superior to placebo if the population difference in response rate is at least 15%. Using the calculations provided by Simon et al. (1985) and accounting for a 20% attrition rate which has been reported in other drug studies using a similar population (Bartzokis et al., 2005), a total sample size of 90 is required, or 45 per treatment group. Since the sample size varies according to the responder rate (as well as the magnitude of the difference of interest), and the responder rate for placebo is unknown, we assume a placebo responder rate of 40%, which is at the high end of the studies referenced above. By contrast, in order to have a one-sided Type I error rate of 5% and 80% power to detect the 15% difference between two

groups using a traditional hypothesis testing framework, the study would require 274~344 participants when the placebo responder rate changes from 20% to 44%, after adjustment for a 20% withdrawal rate. If the true population difference in response rate is smaller than 15% (between 10~12%) and if we still consider it clinical significant, the proposed sample size (n=72 completers or 90 with 20% withdrawal rate) will still provide at least 80~85% probability of correctly identifying the superior group.

4.0. Recruitment and Feasibility

Active recruitment will take place over 31 months. To assess the feasibility of recruiting the proposed number of participants the VHA Support Services Center data files for Special Focus Groups were reviewed to determine how many combat veterans with PTSD currently seek care at each of the potential sites. Questionnaires were sent to the sites listed in Table 3 and investigators were asked about their level of interest, perceived ability to recruit the requisite number of patients, proposed strategies for recruiting unmedicated and stably medicated participants, their previous experience with PTSD trials, and about any potentially competing studies. Based on their responses and ability to use the local IRB, the four 1.0 FTE sites initially chosen were Albuquerque (terminated in February 2015), Bronx, Durham, and San Diego; the two .50 FTE sites chosen are Salisbury and Little Rock (added in April, 2015).

Table 3: Site Selection

VAMC	Number of PTSD Patients	Number of Previous PTSD Trials
Albuquerque	6,594	6
Birmingham	5,289	4
Bronx	1,595	5
Durham	5,775	9
Pittsburgh	1,743	1
Providence	2,647	0
Salt Lake City	3,725	2
San Diego	4,733	3
Temple	6,216	0
Salisbury	5,409	7
Little Rock	4,051	1

Researchers at the Bronx site (PIs Yehuda and Golier) have extensive experience designing and conducting research projects; a study of prolonged exposure therapy at the Bronx site entitled "Neuroendocrine Correlates of PTSD Before and After Treatment" has enrolled 103 participants since May 2006. "Cortisol Augmentation of a Psychological Treatment in Warfighters with PTSD" recruited 32 participants in the past year. Additionally, the Bronx site has a specialized outpatient PTSD clinic, close links with primary care and the ability to extend recruitment to other local VAs (Manhattan, Brooklyn, Northport, Montrose, East Orange) and community-based outpatient clinics.

The team at Durham has a vast amount of experience with randomized controlled medication studies in PTSD and is well versed in performing the assessments and evaluations that will be used in the present study. For CSP 504, Durham was the second site in terms of recruitment (26 participants in 36 months). Additionally, Durham has participated in eight other trials: 1) CSP 563 recruited 13 participants in 15 months; 2) "A Pilot Randomized Controlled Trial in Sub-threshold PTSD with Paroxetine in Veterans who Served after September 11, 2001" recruited 12 participants in one year; 3) "Bupropion SR for Social and Occupational Dysfunction, Symptom Reduction, and Smoking Cessation in PTSD" recruited 30 participants in two years; 4) "A Placebo-Controlled Evaluation of the Effects of Bupropion for Smoking Cessation in Patients with PTSD" recruited 15 participants in one year; 5) "Lamotrigine in the Treatment of PTSD" recruited 8 participants in one year; 6) "Preliminary Study of Nefazodone in PTSD" recruited 10 participants in one year; 7) "Double-Blind Comparison of Sertraline and Placebo in Outpatients with PTSD" recruited 22 participants in two years; and 8) "Fluoxetine Treatment of PTSD" recruited 11 patients in 18 months.

San Diego is an active research site with a large patient population and high retention and completion rates for participants enrolled in clinical trials. The San Diego site PI, Dr. Baker, has been an investigator in seminal pharmacotherapy trials in PTSD including: 1) " A Double-Blind, Randomized, Placebo-Controlled, Multi-Center Study of Brofaromine in the Treatment of PTSD;" 2) "Sertraline Treatment of PTSD: A Randomized Controlled Trial;" and 3) "Randomized, Double-Blind Comparison of Sertraline and Placebo for PTSD in a Department of Veterans Affairs Setting." Additionally, San Diego has participated in three large, multi-site trials: 1) CSP 519 ("Integrating Practice Guidelines for Smoking Cessation Into Mental Health Care for PTSD") recruited 88 participants in 36 months; 2) CSP 504 recruited 19 participants in 25 months; and 3)

“A Randomized, Placebo-Controlled Trial of Nepicastat for the Treatment of PTSD in OIF/OEF Veterans” recruited 9 participants in 11 months.

Albuquerque has been one of the top recruitment sites for two CSP studies in PTSD: CSP 563 (“Prazosin and Combat Trauma”, having recruited 12 participants in 11 months) and CSP 504 (“Risperidone Treatment for Military Service Related Chronic PTSD”, 14 participants in 30 months). Recruitment was also high in other PTSD studies. However due to low recruitment in this study, this site was terminated in March, 2015. Salisbury and Little Rock were added in April 2015, instead, to help recruitment.

Salisbury VAMC was a very successful site for CSP 563 (“Prazosin and Combat Trauma”). It is also home to a MIRECC dedicated to the study of post-deployment mental health problems including PTSD and mTBI. It has a full research infrastructure and ready-access to the study group of interest.

Little Rock VA has just successfully recruited for and completed several studies of PTSD focused on a similar participant pool. One was a randomized controlled trial of modafanil in OEF/OIF combat veterans with PTSD (25 subjects completed the RCT over an 18 month recruiting period). Dr. Kimbrell, the site investigator, was also the collaborating psychiatrist on “Telemedicine-based collaborative care for posttraumatic stress disorder: a randomized clinical trial” and facilitated a study examining psychophysiological measures in OIF/OEF veterans.

We propose to enroll 90 participants across multiple VA sites to yield 72 completers based on an estimated attrition rate of 20%. We expect the 3 active sites (Bronx, San Diego, and Durham) to enroll 25~26 patients each over the course of 31 months (0.8 per site per month); and the 2 added sites (Salisbury and Little Rock) to enroll 5~6 patients each over the course of 9~10 months (0.5~0.6 per site per month). The Albuquerque site that was terminated in February 2015 enrolled a total of 2 participants.

Given the data provided by the VHA Support Services Center and the individual study sites, this recruitment goal, which takes into account the challenges of recruiting unmedicated veterans and stably medicated veterans, is feasible.

4.p. Statistical Analyses

4.p.1. *Analysis Plan*

The randomization will be assessed by comparing the distribution of all baseline characteristics including age, race, marital status, education, duration of symptoms, duration of illness since trauma, overall extent of the psychiatric co-morbidity, whether or not on concurrent psychotropic medications and number of those medications used prior to randomization, baseline total CAPS score, military cohort, and site across the two randomized groups.

Primary Outcome:

Primary Analysis of Primary Outcome -The primary analysis for this study will not be based on a hypothesis test; rather, responder rates at one month will be calculated for each of two groups. If mifepristone 600 mg is observed to have at least a 15% higher responder rate than placebo, then we are at least 90% certain we have detected a clinically relevant signal. This analysis will be performed based on a modified intent-to-treat (mITT) principle. All randomized participants will be included in the analyses except for those for whom there is positive proof that they did not take a single dose of study medication (e.g. medication never picked up, full bottle returned, patient immediately declines participation after study medication has been dispensed) or there is positive proof that they are deemed ineligible after randomization and are terminated in the 7-day treatment phase due to safety concern. For patients who do not have one-month CAPS total score, their responses will be estimated using multiple imputation technique as outlined in Molenberghs and Kenward, 2007, Chapter 9 and implemented in the SAS procedure MI using participants aforementioned baseline characteristics and available information at other time points (baseline, 1 week, 12 week). The imputation will be done within each treatment group.

Secondary Analysis of Primary Outcome -The primary outcome will also be compared using chi-square tests. Additionally, logistic regression models will be used to adjust for aforementioned baseline characteristics. The model selection will use a backward elimination procedure and be verified by a forward selection procedure.

Exploratory Analysis of Primary Outcome -The primary outcome will also be explored for subgroups as defined by concurrent psychotropic medication use status and classification prior to randomization. The mITT principle will also be applied in the secondary and exploratory analyses of the primary outcome.

Sensitivity Analysis of Missing Data- In the analyses mentioned above, missing data will be replaced using three additional procedures. The first procedure will entail the assumption of "worst case scenario" where the participants with missing data at week 4 will be deemed non-responders. This approach will provide a conservative test of treatment effects. In the second procedure, the last available information about the primary outcome, if available at week 1, will be used to impute the missing data at week 4 and if missing at both week 1 and 4, participants will be deemed non-responders. The third procedure involves the comparison on all participants whose CAPS score are obtained on week 4.

<u>Secondary Outcomes:</u>

1. The analysis specified for the one-month responder rate will also be done for the three-month responder rate as a secondary analysis to check for the sustainability of the response.

   Additionally, a continuous mixed-effects model for longitudinal observations of CAPS (past week symptom status) scores (week 1, 4 and 12) will be used. Covariates including baseline characteristics, specifically duration of symptoms, duration of illness since trauma, concurrent psychotropic medication use prior to randomization, overall extent of the psychiatric co-morbidity, military cohort, and site will be explored in the adjusted analysis. This analysis will provide a treatment effect in terms of the slope of the interaction term between group and time variables, conditional on aforementioned covariates under the missing at random (MAR) assumption (Laird and Ware, 1982).

   The above mentioned analyses will be performed based on a mITT principle.

2. To describe the safety and tolerability mifepristone, information on all related and unrelated SAEs will be collected. There will be accelerated safety reporting for every two adverse events in every ten participants. The specific AE(s) leading to the withdrawal of a patient from study participation will be collected, along with characteristics of the AE including severity, duration, and whether the withdrawal was at the request of the patient or was initiated by a study investigator or other clinician. The AE rate will be determined on the basis of occurrence of at least one adverse event in each participant, and the following comparisons will be carried out:

   a. A comparison of individual adverse event rates between mifepristone and placebo.
   b. A comparison of overall adverse event rates between mifepristone and placebo.
   c. A comparison of severity of adverse events (grades 1, 2, 3, 4, and 5) between mifepristone and placebo
   d. A comparison of overall adverse event rates classified as possibly or definitely attributable to study drug, between mifepristone and placebo.
   e. A comparison of overall adverse event rates classified as possibly or definitely attributable to study drug, between mifepristone and placebo by weeks 1, 4 and 12.

Table 4. Adverse Event Rates by Treatment Arm

	Placebo	600mg
Individual AE Rate (AE 1)		
....		
Individual AE Rate (AE k)		
Overall AE Rate		
Overall AE Grade1		
Overall AE Grade 2		
Overall AE Grade 3		
Overall AE Grade 4		

Overall AE Grade 5		
Overall AE not attributable to study drug		
Overall AE (possibly or definitely) attributable to study drug		

Table 5. Adverse Event Rates by Study Visits and Treatment Arm

	AEs Received by Week 1		AEs Received after Week 1 till Week 4		AEs Received After Week 4 till Week 12	
	PBO	600 mg	PBO	600 mg	PBO	600 mg
Individual AE Rate (AE 1)						
....						
Individual AE Rate (AE k)						
Overall AE Rate						
Overall AE Grade1						
Overall AE Grade 2						
Overall AE Grade 3						
Overall AE Grade 4						
Overall AE Grade 5						
Overall AE not attributable to study drug						
Overall AE (possibly or definitely) attributable to study drug						

The safety analysis will be performed based on safety population. All randomized patients who have received at least one dose of study medication will be included in the safety analysis according to the treatment groups to which they are randomized.

Descriptive Outcomes:

Descriptive statistics and confidence intervals for the change from baseline to one month follow-up and to three month follow-up for each of the two treatment groups will be provided for each of the descriptive outcome measures. Additionally, comparisons between mifepristone and placebo will be performed; however, lack of statistical significance should not be construed as evidence of no clinical effect given the small sample size. All efficacy analyses on descriptive outcomes will be performed based on a mITT principle.

1. PTSD Symptom Sub-Scales
   a. Descriptive statistics for the change in score from baseline to one month on the three PTSD symptom sub-scales (intrusions, hyperarousal, and avoidance) and confidence intervals will be provided for each treatment group.
   b. Change in score from baseline to one month on the three PTSD symptom sub-scales (intrusions, hyperarousal, and avoidance) will be compared between mifepristone and placebo using two-sample t-test (or Wilcoxon rank-sum test if the normality assumption does not hold).

2. Depression
   a. Descriptive statistics for the change in score from baseline to one month on the Beck Depression Inventory (BDI) and confidence intervals will be provided for each treatment group.
   b. Change in score from baseline to one month on the Beck Depression Inventory (BDI) will be compared between mifepristone and placebo using two-sample t-test (or Wilcoxon rank-sum test if the normality assumption does not hold).

3. PTSD Symptom Severity

a. Descriptive statistics for the change in score from baseline to one month on the PTSD Checklist (PCL) and confidence intervals will be provided for each treatment group.
b. Change in score from baseline to one month on the PTSD Checklist (PCL) will be compared between mifepristone and placebo using two-sample t-test (or Wilcoxon rank-sum test if the normality assumption does not hold).

4. Sleep Quality
   a. Descriptive statistics for the change in score from baseline to one month on the Pittsburgh Sleep Quality Index (PSQI) and confidence intervals will be provided for each treatment group.
   b. Change in score from baseline to one month on the Pittsburgh Sleep Quality Index (PSQI) will be compared between mifepristone and placebo using two-sample t-test (or Wilcoxon rank-sum test if the normality assumption does not hold).

5. Anger
   a. Descriptive statistics for the change in score from baseline to one month on the STAXI and confidence intervals will be provided for each treatment group.
   b. Change in score from baseline to one month on the STAXI will be compared between mifepristone and placebo using two-sample t-test (or Wilcoxon rank-sum test if the normality assumption does not hold).

6. Functional Impairment
   a. Descriptive statistics for the change in score from baseline to one month on the functional impairment measure (item 24) from the CAPS and confidence intervals will be provided for each treatment group.
   b. Change in score from baseline to one month on the functional impairment measure (item 24) from the CAPS will be compared between mifepristone and placebo using two-sample t-test (or Wilcoxon rank-sum test if the normality assumption does not hold).

7. Neuroendocrine
   a. Descriptive statistics for the change in score from baseline to one week following treatment, and baseline to one month and confidence intervals for plasma cortisol and ACTH levels will be provided for each treatment group.
   b. Change in plasma cortisol and ACTH levels from baseline to one week following treatment and baseline to one month will be compared between mifepristone and placebo using two-sample t-test (or Wilcoxon rank-sum test if the normality assumption does not hold).
   c. Mean plasma cortisol levels at one month will be compared between clinical responders (30% reduction in CAPS score) and non-responders using a t-test (or Wilcoxon rank-sum test if the normality assumption does not hold). This analysis will be limited to those on mifepristone only.
   d. Mean ACTH levels at one month will be compared between clinical responders (30% reduction in CAPS score) and non-responders using a t-test (or Wilcoxon rank-sum test if the normality assumption does not hold). This analysis will be limited to those on mifepristone only.

8. Mifepristone and its metabolites
   a. Descriptive statistics and confidence intervals for one week following treatment for plasma levels of mifepristone and its metabolites will be provided.
   b. Mean plasma levels of mifepristone and it metabolites at one week will also be compared between clinical responders (30% reduction in CAPS score) and non-responders using a t-test (or Wilcoxon rank-sum test if the normality assumption does not hold).
   c. A continuous mixed-effects model for longitudinal observations of CAPS scores will also be used adjusting for aforementioned baseline characteristics, indicator variable that contrasts

mifepristone vs. placebo, and plasma levels of mifepristone and it metabolites at one week to determine the effect of them on CAPS scores trajectories.

Although it is not listed as an outcome, the percentage of unique participants with concurrent psychotropic medication use change by 1 month and by 3 month (end of the trial) will be compared between mifepristone and placebo using chi-square tests. Concurrent psychotropic medication change is defined as seeking a rescue medication for those who are not on concurrent psychotropic medication prior to randomization, or seek a new psychotropic medication or require a dose increase for those on a stabilized regimen treating PTSD prior to randomization. Those analyses will also be based on mITT principle. Except the primary analysis of the primary outcome, all efficacy and safety analyses will be performed with a significance level of 5% for two-sided tests. Due to exploratory nature of the study, and the sample size was not powered on hypothesis testing frame work, careful consideration is necessary when interpreting the significance of these results on the primary, secondary and descriptive outcomes. The results of the trial are considered to be hypothesis generating and are not to obtain definitive efficacy information. No adjustments for multiplicity on various secondary outcome measures are planned in this phase IIa trial. The lack of adjustment will be noted in the resulting manuscripts.

Description of Protocol Drugs:

This is a Phase IIa trial of a medication that is approved by the FDA for termination of pregnancy, which results from its anti-progesterone activity and for the treatment of hyperglycemia in Cushing's Syndrome in patients who have failed surgery or are not candidates for surgery. It is being tested in this study since it is also an effective glucocorticoid receptor antagonist. This will be the initial trial examining the effects of mifepristone in PTSD. The CCTA study pharmacist will file an Investigational New Drug Application (IND) with the FDA, and if the IND is accepted by FDA, will serve as the Sponsor's Representative for the study. Mifepristone (Korlym®) 300 mg tablets and matching placebo tablets will be supplied to the study sites by the CSP Clinical Research Pharmacy Coordinating Center (PCC), packaged in bottles with safety caps. Each bottle will contain a seven-day supply as follows: 600 mg dose =fourteen 300-mg active mifepristone tablets; placebo dose = fourteen matching placebo tablets. Only one bottle will be required for each participant for the week of treatment, and will be labeled with a tracking identification number tied to a randomization number. No extra tablets will be included in the bottles. A supply of emergency bottles will be available at the sites for lost or damaged bottles, and will be assigned by contacting the PCC. Two tablets will be taken orally every evening with a meal. Participants who drop or in some way make unusable one or more tablets for a specific daily dose will be instructed to contact their site investigator the next morning. Adverse medical events will be assessed as outlined in the section on monitoring adverse events. Unused and expired drug will be disposed of as directed by the PCC.

Laboratory Evaluations:

Basal plasma cortisol and ACTH**:** For endocrine testing veterans will be asked to fast from midnight the night before the blood draw. Blood samples for hormonal analysis are collected into tubes containing ethylenediaminetetraacetic acid (EDTA), spun immediately in a cold centrifuge, and frozen for subsequent analysis. Since observed changes in ACTH and cortisol could effectively break the blind, samples will be assayed at the Clinical Neuroscience Lab at the James J. Peters VA Medical Center (Director: Dr. Yehuda).

Plasma ACTH levels will be assayed using ELISA kits from ALPCO Diagnostics (Windham, NH). The detection limit is 0.5 pg/ml. The intra- and inter-assay variability is 3.7% and 6.0% respectively.

Mifepristone and metabolites: Plasma samples will be obtained for assessment of mifepristone and its metabolites (RU 42633, RU 42698, and RU 42848) at immediate follow-up. Samples will be frozen at the sites and shipped in batches to MicroConstants, Inc. (San Diego, CA) where they will be assayed.

Risk/Benefit Assessment:

Mifepristone: Mifepristone is a generally safe and tolerable drug that has been studied before in persons with neuropsychiatric disorders. Serious adverse effects have occurred when it is used for outpatient pregnancy termination (e.g., hemorrhage, shock, sepsis, death), but these are related to the procedures and effects of medically induced abortion, not the toxicity of the drug. In short-term studies, mifepristone has been

associated with a body rash which resolved when the medication is discontinued. Mifepristone use has also been associated with nausea, fatigue, headache, hot flashes, and breast tenderness in long-term trials in medical diseases. There is a remote chance that mifepristone, by blocking the effects of the stress hormone cortisol, could cause functional adrenal insufficiency; therefore, persons with adrenal insufficiency or with low baseline cortisol levels will be excluded. Additionally, patients will be monitored for signs and symptoms of adrenal insufficiency during the trial (severe fatigue, weakness, nausea, vomiting). Patients who have renal disease/impairment, hepatic disease/impairment, hypokalemia, and prolonged QTc interval>450 msec on ECG at screening will also be excluded. Patients taking simvastatin, lovastatin, fentanyl, pimozide, bupropion, nefazodone, dihydroergotamine, ergotamine, quinidine, sirolimus, alprazolam, or tacrolimus have an increased risk of adverse events associated with mifepristone use; therefore, participants who receive these drugs will also be excluded. Since grapefruit juice may increase the amount of mifepristone in the blood and may increase the chance of side effects, participants will be instructed to not drink grapefruit juice while they are taking study medication. Other efforts have been made to further reduce the risks associated with mifepristone, such as excluding patients with diabetes and other major medical illnesses, and monitoring of laboratory values, vital signs, and clinical state during the trial. Based on mifepristone's extensive hepatic metabolism by CYP3A4, it is possible that potent inhibitors of this enzyme (e.g., fluconazole, ketoconazole, itraconazole, and erythromycin) could increase mifepristone's blood levels and decrease CYP3A4-mediated generation of its active metabolites. Furthermore, rifampin and certain anticonvulsants (e.g., phenytoin, phenobarbital, and carbamazepine) can considerably accelerate mifepristone's metabolism, decreasing the levels of both mifepristone and its metabolites. Because such interactions have the potential to significantly impact safety and response levels, patients who need to receive strong CYP3A4 inducers or inhibitors as maintenance therapy will be excluded from the study. In addition, randomization will not take place until at least five of these agents' half-lives have expired.

Potential benefits: Since there are few effective treatments for PTSD in veterans, identification of an effective treatment or of a biological target for treatment would be very beneficial; the medication has been found to be safe and tolerable in other neuropsychiatric conditions; thus, the overall risk/benefit ratio is favorable.

Reference:

Aerni A, Traber R, Hock C, Roozendaal B, Schelling G, Papassotiropoulos A, Nitsch RM, Schnyder U, de Quervain DJ. Low-dose cortisol for symptoms of posttraumatic stress disorder. Am J Psychiatry. 2004 Aug;161(8):1488-90.

Baker DG,West SA, NicholsonWE, Ekhator NN, Kasckow JW, Hill KK, Bruce AB, Orth DN, Geracioti TD Jr. Serial CSF corticotropin-releasing hormone levels and adrenocortical activity in combat veterans with posttraumatic stress disorder. Am J Psychiatry. 1999 Apr;156(4):585-8.

Baker DG, Ekhator NN, Kasckow JW, Dashevsky B, Horn PS, Bednarik L, Geracioti TD Jr. Higher levels of basal serial CSF cortisol in combat veterans with PTSD Am J Psychiatry. 2005; 162(5):992-4.

Bartzokis G, Lu PH, Turner J, Mintz J, Saunders CS. Adjunctive risperidone in the treatment of chronic combat-related posttraumatic stress disorder. Biol Psychiatry. 2005 Mar 1;57(5):474-9.

Beck AT, Steer RA, Brown GK. Beck Depression Inventory-II San Antonio, TX: Psychological Corp. 1996,8:229-242.

Belanoff JK, Flores BH, Kalezhan M, Sund B, Schatzberg AF. Rapid reversal of psychotic depression using mifepristone. J Clin Psychopharmacol. 2001 Oct;21(5):516-21.

Belanoff JK, Rothschild AJ, Cassidy F, DeBattista C, Schold C, Schatzberg AF. An open label trial of mifepristone for psychotic major depression. Biol Psychiatry. 2002 1;52(5):386-92.

Bell JB, Nye EC. Specific symptoms predict suicidal ideation in Vietnam combat veterans with chronic posttraumatic stress disorder. Mil Med. 2007 Nov;172(11):1144-7.

Bertagna X, Bertagna C, Luton JP, Husson JM, Girard F. The new steroid analog RU 486 inhibits glucocorticoid action in man J Clin Endocrinol Metab. 1984 Jul;59(1):25-8.

Bertagna X, Escourolle H, Pinquier JL, Coste J, Raux-Demay MC, Perles P, Silvestre L, Luton JP, Strauch G. Administration of RU 486 for 8 days in normal volunteers: antiglucocorticoid effect with no evidence

of peripheral cortisol deprivation. J Clin Endocrinol Metab. 1994 Feb;78(2):375-80.
Blake DD,Weathers FW, Nagy LM, Kaloupek DG, Gusman FD, Charney DS, Keane TM. The development of a Clinician-Administered PTSD Scale. J Trauma Stress. 1995 8(1):75-90.
Blasey CM, Debattista C, Roe R, Belanoff JK. A multisite trial of mifepristone for the treatment of psychotic depression: Contemp Clin Trials. 2009 30(4):284-8.
Boscarino JA. Psychobiologic predictors of disease mortality after psychological trauma: implications for research and clinical surveillance. J Nerv Ment Dis. 2008 196(2):100-7.
Brady K, Pearlstein T, Asnis GM, Baker D, Rothbaum B, Sikes CR, Farfel GM. Efficacy and safety of sertraline treatment of posttraumatic stress disorder: a randomized controlled trial. JAMA. 2000 Apr. 12;283(14):1837-44.
Bremner JD, Southwick SM, Darnell A, Charney DS. Chronic PTSD in Vietnam combat veterans: course of illness and substance abuse. Am J Psychiatry. 1996 Mar;153(3):369-75.
Bremner JD, Licinio J, Darnell A, Krystal JH, Owens MJ, Southwick SM, Nemeroff CB, Charney DS. Elevated CSF CRF concentrations in PTSD. Am J Psychiatry. 1997;154(5):624-9.
Bremner JD, Vythilingam M, Vermetten E, Southwick SM, McGlashan T, Nazeer A, Khan S, Vaccarino LV, Soufer R, Garg PK, Ng CK, Staib LH, Duncan JS, Charney DS. MRI and PET study of deficits in hippocampal structure and function in women with childhood sexual abuse and posttraumatic stress disorder. Am J Psychiatry. 2003 May;160(5):924-32.
Bremner JD, Vythilingam M, Vermetten E, Afzal N, Nazeer A, Newcomer JW, Charney DS. Effects of dexamethasone on declarative memory function in posttraumatic stress disorder. Psychiatry Res. 2004 Nov 30;129(1):1-10.
Buckley T, Duggal V, Schatzberg AF. The acute and post-discontinuation effects of a glucocorticoid receptor (GR) antagonist probe on sleep and the HPA axis in chronic insomnia: a pilot study. J Clin Sleep Med. 2008 Jun 15;4(3):235-41.
Buysse DJ, Reynolds CF 3rd, Monk TH, Berman SR, Kupfer DJ. The Pittsburgh Sleep Quality Index: a new instrument for psychiatric practice and research. Psychiatry Res. 1989 May;28(2):193-213. Davidson JR, Rothbaum BO, van der Kolk BA, Sikes CR, Farfel GM. Multicenter, double-blind comparison of sertraline and placebo in the treatment of posttraumatic stress disorder. Arch Gen Psychiatry. 2001 May;58(5):485-92.
De Kloet ER, Vreugdenhil E, Oitzl MS, Joëls M. Brain corticosteroid receptor balance in health and disease. Endocr Rev. 1998 Jun;19(3):269-301. Review.
De Kloet ER, Derijk R. Signaling pathways in brain involved in predisposition and pathogenesis of stressrelated disease: genetic and kinetic factors affecting the MR/GR balance. Ann N Y Acad Sci. 2004 Dec; 1032:14-34. Review.
DeBattista C, Belanoff J, Glass S, Khan A, Horne RL, Blasey C, Carpenter LL,Alva G. Mifepristone versus Placebo in the Treatment of Psychosis in Patients withPsychotic Major Depression.Biol Psychiatry. 2006 Dec 15;60(12):1343-9.
Flores BH, Kenna H, Keller J, Solvason HB, Schatzberg AF. Clinical and biological effects of mifepristone treatment for psychotic depression. Neuropsychopharm. 2006 31(3):628-36.
Friedman MJ, Marmar CR, Baker DG, Sikes CR, Farfel GM. Randomized, double-blind comparison of sertraline and placebo for PTSD in a Department of Veterans Affairs setting. J Clin Psychiatry. 2007 May;68(5):711-20.
Gaillard RC, Riondel A,Muller AF, HerrmannW, Baulieu EE. RU 486: a steroid with antiglucocorticosteroid activity that only disinhibits the human pituitary-adrenal system Proc Natl Acad Sci U S A. 1984 Jun;81(12):3879-82.
Gallagher P,Watson S, Smith MS, Ferrier IN, Young AH. Effects of adjunctive mifepristone (RU-486) administration on neurocognitive function and symptoms in schizophrenia. Biol Psychiatry. 2005 Jan 15;57(2):155-61.
Gallagher P,Watson S, Elizabeth Dye C, Young AH, Nicol Ferrier I. Persistent effects of mifepristone (RU-486) on cortisol levels in bipolar disorder and schizophrenia. J Psychiatr Res. 2008 Oct;42(12):1037-41.
Gibbons JD, Olkin I, Sobel M. (1997). Selecting and ordering populations: a new statistical methodology. John

Wiley and Sons, Inc., New York.

Gilbertson MW, Gurvits TV, Lasko NB, Orr SP, Pitman RK. Multivariate assessment of explicit memory function in combat veterans with posttraumatic stress disorder. J Trauma Stress. 2001 Apr;14(2):413-32.

Gilbertson MW, ShentonME, Ciszewski A, Kasai K, Lasko NB, Orr SP, Pitman RK. Smaller hippocampal volume predicts pathologic vulnerability to psychological trauma. Nat Neurosci. 2002 Nov;5(11):1242-7.

Golier JA, Yehuda R, Lupien SJ, Harvey PD. Memory for trauma-related information in Holocaust survivors with PTSD. Psychiatry Res. 2003 Dec 1;121(2):133-43.

Griffin MG, Resick PA, Yehuda R. Enhanced cortisol suppression following dexamethasone administration in domestic violence survivors.Am J Psychiatry. 2005 Jun;162(6):1192-9.

Gurvits TV, Shenton ME, Hokama H, Ohta H, Lasko NB, Gilbertson MW, Orr SP, Kikinis R, Jolesz FA, McCarley RW, Pitman RK. MRI study of hippocampal volume in chronic, combat-related posttraumatic stress disorder. Biol Psychiatry. 1996 Dec 1;40(11):1091-9.

Heikinheimo O, Kekkonen R, Lähteenmäki P. The pharmacokinetics of mifepristone in humans reveals insights into differential mechanisms of antiprogestin action. Contraception. 2003 Dec;68(6):421-6.

Hertzberg MA, Feldman ME, Beckham JC, Kudler HS, Davidson JR. Lack of efficacy for fluoxetine in PTSD in combat veterans. Ann Clin Psychiatry. 2000;12(2):101-5.

Institute of Medicine (IOM). 2008. Treatment of posttraumatic stress disorder: An assessment of the evidence.Washington, DC: The National Academies Press.

Jakupcak M, Cook J, Imel Z, Fontana A, Rosenheck R, McFall M. Posttraumatic stress disorder as a risk factor for suicidal ideation in Iraq and AfghanistanWar veterans. J Trauma Stress. 2009 Aug;22(4):303-6.

Kanter ED,Wilkinson CW, Radant AD, Petrie EC, Dobie DJ, McFall ME, Peskind ER, Raskind MA. Glucocorticoid feedback sensitivity and adrenocortical responsiveness in posttraumatic stress disorder. Biol Psychiatry. 2001 Aug 15;50(4):238-45.

Karssen AM, Belanoff JK, De Kloet ER. 2003. Glucocorticoid receptor antagonist C-1073 (mifepristone/RU486) inhibits P-glycoprotein-mediated efflux transport of cortisol. XXXIV congress of International Society of Psychoneuroendocrinology, New York, USA, Sept 7-9 (Abstract).

Klein JP, MoeschbergerML. Survival analysis: Techniques for censored and truncated data. Springer-Verlag, New York Inc. 2003.

Kosten TR, Mason JW, Giller EL, Ostroff RB, Harkness L. Sustained urinary norepinephrine and epinephrine elevation in post-traumatic stress disorder. Psychoneuroendocrinology. 1987;12(1):13-20.

Kulka RA, SchlengerWE, Fairbank J. 1990. Trauma and the VietnamWar Generation.Bruner-Mazel, Bruner-Mazel. Laird NM,Ware JH. Random effects models for longitudinal data. Biometrics. 1982, 38: 963-74.

Laue L, Lotze MT, Chrousos GP, Barnes K, Loriaux DL, Fleisher TA. Effect of chronic treatment with the glucocorticoid antagonist RU 486 in man:toxicity, immunological, and hormonal aspects. J Clin Endocrinol Metab. 1990 Dec;71(6):1474-80.

Liberzon I, Taylor SF, Amdur R, Jung TD, Chamberlain KR, Minoshima S, Koeppe RA, Fig LM. Brain activation in PTSD in response to trauma-related stimuli. Biol Psychiatry. 1999 Apr 1;45(7):817-26.

Lupien SJ, Gillin CJ, Hauger RL.Working memory is more sensitive than declarative memory to the acute effects of corticosteroids in humans. Behav Neurosci. 1999 Jun;113(3):420-30.

Martenyi F, Brown EB, Zhang H, Prakash A, Koke SC. Fluoxetine versus placebo in posttraumatic stress disorder. J Clin Psychiatry. 2002 Mar;63(3):199-206.

Marshall RD, Beebe KL, Oldham M, Zaninelli R. Efficacy and safety of paroxetine treatment for chronic PTSD: a fixed-dose, placebo-controlled study. Am J Psychiatry. 2001 Dec;158(12):1982-8.

Mohamed S, Rosenheck RA. Pharmacotherapy of PTSD in the U.S. Department of Veterans Affairs: diagnostic- and symptom-guided drug selection. J Clin Psychiatry. 2008 Jun;69(6):959-65.

Molenberghs G, Kenward MG. Missing data in clinical studies, JohnWiley & Sons Ltd.,West Sussex, England 2007.

Newport DJ, Heim C, Bonsall R, Miller AH, Nemeroff CB. Pituitary-adrenal responses to standard and lowdose dexamethasone suppression tests in adult survivors of child abuse.Biol Psychiatry. 2004 Jan 1;55(1):10-20.

Neylan TC, Schuff N, Lenoci M, Yehuda R,WeinerMW, Marmar CR. Cortisol levels are positively correlated

with hippocampal N-acetylaspartate.Biol Psychiatry. 2003 Nov 15;54(10):1118-21.

Oitzl MS, Fluttert M, SutantoW, de Kloet ER. Continuous blockade of brain glucocorticoid receptors facilitates spatial learning and memory in rats. Eur J Neurosci. 1998 Dec;10(12):3759-66.

Pitman RK. Post-traumatic stress disorder, hormones, and memory. Biol Psychiatry. 1989 Jul;26(3):221-3. Review.

Posner K, Brent D, Lucas C, Gould M, Stanley B, Brown G, Fisher P, Zelazny J, Burke A, Oquendo M, Mann J. 2008. The Columbia Suicide Severity Rating Scale. Columbia University: New York, NY.

Raison CL, Miller AH.When not enough is too much: the role of insufficient glucocorticoid signaling in the pathophysiology of stress-related disorders. Am J Psychiatry. 2003 Sep;160(9):1554-65.

Rasmusson AM, Lipschitz DS,Wang S, Hu S, Vojvoda D, Bremner JD, Southwick SM,Charney DS. Increased pituitary and adrenal reactivity in premenopausal women with posttraumatic stress disorder. Biol Psychiatry. 2001 Dec 15;50(12):965-77.

Rauch SL, Shin LM, Phelps EA. Neurocircuitry models of posttraumatic stress disorder and extinction: human neuroimaging research--past, present, and future. Biol Psychiatry. 2006 Aug 15;60(4):376-82. Review.

Raux-Demay MC, Pierret T, Bouvier d'Yvoire M, Bertagna X, Girard F. Transient inhibition of RU 486 antiglucocorticoid action by dexamethasone. J Clin Endocrinol Metab. 1990 Jan;70(1):230-3.

Sarkar, N.N. Bioavailability, Pharmacokinetics, and Use-Effectiveness. Eur J Obstet Gynecol Reprod Biol. 2002 Mar 10;101(2):113-20.

Schelling G, Roozendaal B, De Quervain DJ. Can posttraumatic stress disorder be prevented with glucocorticoids? Ann N Y Acad Sci. 2004 Dec; 1032: 158-66.

Schelling G, Roozendaal B, Krauseneck T, Schmoelz M, DE Quervain D, Briegel J. Efficacy of hydrocortisone in preventing posttraumatic stress disorder following critical illness and major surgery. Ann N Y Acad Sci. 2006 Jul; 1071: 46-53. Review.

Seal KH, Metzler TJ, Gima KS, Bertenthal D, Maguen S, Marmar CR. Trends and Risk Factors forMental Health Diagnoses Among Iraq and Afghanistan Veterans Using Department of Veterans Affairs Health Care, 2002-2008. Am J Public Health. 2009 Jul 16.

Seedat S,Warwick J, van Heerden B, Hugo C, Zungu-Dirwayi N, Van Kradenburg J,Stein DJ. Single photon emission computed tomography in posttraumatic stress disorder before and after treatment with a selective serotonin reuptake inhibitor. J Affect Disord. 2004 May;80(1):45-53.

Shin LM,Wright CI, Cannistraro PA,Wedig MM,McMullin K, Martis B, Macklin ML, Lasko NB, Cavanagh SR, Krangel TS, Orr SP, Pitman RK,Whalen PJ, Rauch SL. A functional magnetic resonance imaging study of amygdala and medial prefrontal cortex responses to overtly presented fearful faces in posttraumatic stress disorder. Arch Gen Psychiatry. 2005 Mar;62(3):273-81.

Simon R,Wittes RE, Ellenberg SS. Randomized phase II clinical trials. Cancer Treatment Report. 1985 Dec;69(12):1375-81.

Simpson GM, El Sheshai A, Loza N, Kingsbury SJ, Fayek M, Rady A, FawzyW. An 8-week open-label trial of a 6-day course of mifepristone for the treatmentof psychotic depression.J Clin Psychiatry. 2005 May;66(5):598-602.

Southwick SM, Krystal JH, Bremner JD, Morgan CA 3rd, Nicolaou AL, Nagy LM, Johnson DR, Heninger GR, Charney DS. Noradrenergic and serotonergic function in posttraumatic stress disorder. Arch Gen Psychiatry. 1997 Aug;54(8):749-58.

Spoont MR, Murdoch M, Hodges J, Nugent S. Treatment receipt by veterans after a PTSD diagnosis in PTSD, mental health, or general medical clinics. Psychiatr Serv. 2010 Jan;61(1):58-63.

Stein MB, Koverola C, Hanna C, Torchia MG, McClarty B. Hippocampal volume in women victimized by childhood sexual abuse. Psychol Med. 1997 Jul;27(4):951-9.

Stein MB, Kennedy CM, Twamley EW. Neuropsychological function in female victims of intimate partner violence with and without posttraumatic stress disorder. Biol Psychiatry. 2002 Dec 1;52(11):1079-88.

Tucker P, Zaninelli R, Yehuda R, Ruggiero L, Dillingham K, Pitts CD. Paroxetine in the treatment of chronic posttraumatic stress disorder: results of a placebo-controlled, flexible-dosage trial. J Clin Psychiatry. 2001 Nov;62(11):860-8.

Van Zuiden M, Geuze E,Willemen HL, Vermetten E, Maas M, Heijnen CJ, Kavelaars A. Pre-existing high glucocorticoid receptor number predicting development of posttraumatic stress symptoms after

military deployment. Am J Psychiatry. 2011 Jan;168(1):89-96.

Von Känel R, Hepp U, Kraemer B, Traber R, Keel M, Mica L, Schnyder U. Evidence for low-grade systemic proinflammatory activity in patients with posttraumatic stress disorder. J Psychiatr Res. 2007 Nov;41(9):744-52.

Vasterling JJ, Brailey K, Constans JI, Sutker PB. Attention and memory dysfunction in posttraumatic stress disorder. Neuropsychology. 1998 Jan;12(1):125-33.

Veazey CH,Wagner AL, Hays JR, Miller HA. Validity of the Miller forensic assessment of symptoms test in psychiatric inpatients. Psychol Rep. 2005 Jun;96(3 Pt 1):771-4.

Vermetten E, Vythilingam M, Schmahl C, DE Kloet C, Southwick SM, Charney DS, Bremner JD. Alterations in stress reactivity after long-term treatment with paroxetine in women with posttraumatic stress disorder. Ann N Y Acad Sci. 2006 Jul; 1071:184-202.

Weathers FW, Litz BT, Keane TM, Herman DS, Steinberg HR, Huska JA, Kraemer HC. The utility of the SCL-90-R for the diagnosis of war-zone related posttraumatic stress disorder. J Trauma Stress. 1996 Jan;9(1):111-28.

Yehuda R, Southwick SM, Krystal JH, Bremner D, Charney DS, Mason JW. Enhanced suppression of cortisol following dexamethasone administration in posttraumatic stress disorder. Am J Psychiatry. 1993 Jan;150(1):83-6.

Yehuda R. Current status of cortisol findings in post-traumatic stress disorder. Psychiatr Clin North Am. 2002 Jun;25(2):341-68, vii.

Yehuda R, Halligan SL, Grossman R, Golier JA,Wong C. The cortisol and glucocorticoid receptor response to low dose dexamethasone administration in aging combat veterans and holocaust survivors with and without posttraumatic stress disorder. Biol Psychiatry. 2002 Sep 1;52(5):393-403.

Yehuda R, Yang RK, Guo SL, Makotkine I, Singh B. Relationship between dexamethasone-inhibited lysozyme activity in peripheral mononuclear leukocytes and the cortisol and glucocorticoid receptor response to dexamethasone. J Psychiatr Res. 2003 Nov-Dec;37(6):471-7.

Yehuda R, Golier JA, Halligan SL, Meaney M, Bierer LM. The ACTH response to dexamethasone in PTSD. Am J Psychiatry. 2004 Aug;161(8):1397-403.

Yehuda R. Status of glucocorticoid alterations in post-traumatic stress disorder. Ann N Y Acad Sci. 2009 Oct; 1179:56-69. Review.

Yehuda R, Flory JD, Pratchett LC, Buxbaum J, Ising M, Holsboer F. Putative biological mechanisms for the association between early life adversity and the subsequent development of PTSD. Psychopharmacology (Berl). 2010 Oct;212(3):405-17.

Young AH, Sahakian BJ, Robbins TW, Cowen PJ. The effects of chronic administration of hydrocortisone on cognitive function in normal male volunteers. Psychopharmacology (Berl) 1999 Aug;145(3):260-6.

Young AH, Gallagher P,Watson S, Del-Estal D, Owen BM, Ferrier IN. Improvements in neurocognitive function and mood following adjunctive treatment with mifepristone (RU-486) in bipolar disorder.Neuropsychopharmacology. 2004 Aug;29(8):1538-45.

Zohar J, Amital D, Miodownik C, Kotler M, Bleich A, Lane RM, Austin C. Double-blind placebo-controlled pilot study of sertraline in military veterans with posttraumatic stress disorder. J Clin Psychopharmacol. 2002 Apr;22(2):190-5.
