# Supplementary material for: Efficacy and Safety of Mifepristone in the Treatment of Male US Veterans With Posttraumatic Stress Disorder: A Phase 2a Randomized Clinical Trial
Source: JAMA Netw Open. 2023 May 9;6(5):e2310223. doi: 10.1001/jamanetworkopen.2023.10223 (PMC10170341; doi:10.1001/jamanetworkopen.2023.10223)
Supplement: Supplement 3. — Data Sharing Statement [file jamanetwopen-e2310223-s003.pdf]

# Data Sharing Statement

Golier. Efficacy and Safety of Mifepristone in the Treatment of Male US Veterans With Posttraumatic Stress Disorder. *JAMA Netw Open*. Published May 09, 2023. doi:10.1001/jamanetworkopen.2023.10223

**Data**
**Data available:** No
